# Innovative Approaches Against Planktonic and Biofilm-Producing *Clostridium perfringens* Associated with Necrotic Enteritis

**DOI:** 10.3390/vetsci13090882

**Published:** 2026-08-28

**Authors:** Ismail Amin, Adel Abdelkhalek, Ioan Pet, Mirela Ahmadi, Norhan K. Abd El-Aziz

**Affiliations:** 1 Microbiology and Parasitology Department, Faculty of Veterinary Medicine, Badr University in Cairo, Badr City 11829, Egypt; 2 Food Safety, Hygiene and Technology Department, Faculty of Veterinary Medicine, Badr University in Cairo, Badr City 11829, Egypt; adel.abdelkhalek@buc.edu.eg; 3 Department of Biotechnology, Faculty of Bioengineering of Animals Resources, University of Life Sciences “King Mihai I” from Timisoara, 300645 Timisoara, Romania; mirelaahmadi@usvt.ro; 4 Department of Microbiology, Faculty of Veterinary Medicine, Zagazig University, Zagazig 44511, Egypt

**Keywords:** *C. perfringens*, antimicrobial resistance, biofilm, toxins, alternative therapy

## Abstract

Necrotic enteritis (NE), caused by the Gram-positive, anaerobic bacterium *Clostridium perfringens* (*C. perfringens*; CP), is one of the most economically important diseases affecting the poultry industry worldwide. The pathogenicity of *C. perfringens* is attributed to the production of more than twenty extracellular toxins and enzymes, with strains classified into seven toxinotypes (A–G) based on their major toxin genes. While the alpha-toxin gene (*cpa*) is present across all *C. perfringens* toxinotypes, *netB* is recognized as the primary virulence determinant of avian NE. However, toxin-gene carriage alone does not establish toxin expression or disease causation. *Cpb2* and *tpeL* have also been associated with virulence in some *C. perfringens* isolates, although their independent contributions to NE pathogenesis remain less firmly established. The increasing prevalence of antimicrobial-resistant *C. perfringens* strains, including multidrug-resistant (MDR), extensively drug-resistant (XDR), and pandrug-resistant (PDR) isolates, together with the organism’s ability to form biofilms, has complicated disease prevention and treatment by enhancing environmental persistence and tolerance to antimicrobial agents. Distinct from reviews that primarily address NE pathogenesis or antibiotic alternatives as broad terms, this review integrates antimicrobial resistance with planktonic and biofilm-associated *C. perfringens*. Furthermore, it critically evaluates alternative interventions based on their activity against these bacterial states and the strength of supporting in vitro, in vivo, and field evidence. In conclusion, this review highlights the urgent need for sustainable, antibiotic-free strategies to combat NE. Advancing the development and field validation of these alternative approaches may contribute to reducing antimicrobial resistance, limiting biofilm-associated persistence, and improving poultry health and productivity.

## Simple Summary

Necrotic enteritis (NE) is an important bacterial disease affecting poultry and causes major economic losses worldwide. It is caused by *Clostridium perfringens* (*C. perfringens*), an aerotolerant anaerobic bacterium that produces many harmful toxins, most notably NetB and alpha-toxin, and forms protective biofilms that help it survive in poultry environments and resist antimicrobial treatments. The steady emergence of antimicrobial-resistant strains has made controlling this disease increasingly challenging. This review summarizes the epidemiology and pathogenicity of *C. perfringens* associated with NE, and discusses promising antimicrobial-free approaches for its control. Particular attention is given to strategies targeting both planktonic and biofilm-forming cells, including their effectiveness under in vitro, in vivo, and field conditions. The review highlights the need for sustainable and effective alternatives to conventional antimicrobials to control NE, reduce antimicrobial resistance, and improve poultry health and production.

## 1. Introduction

**Figure 1:**
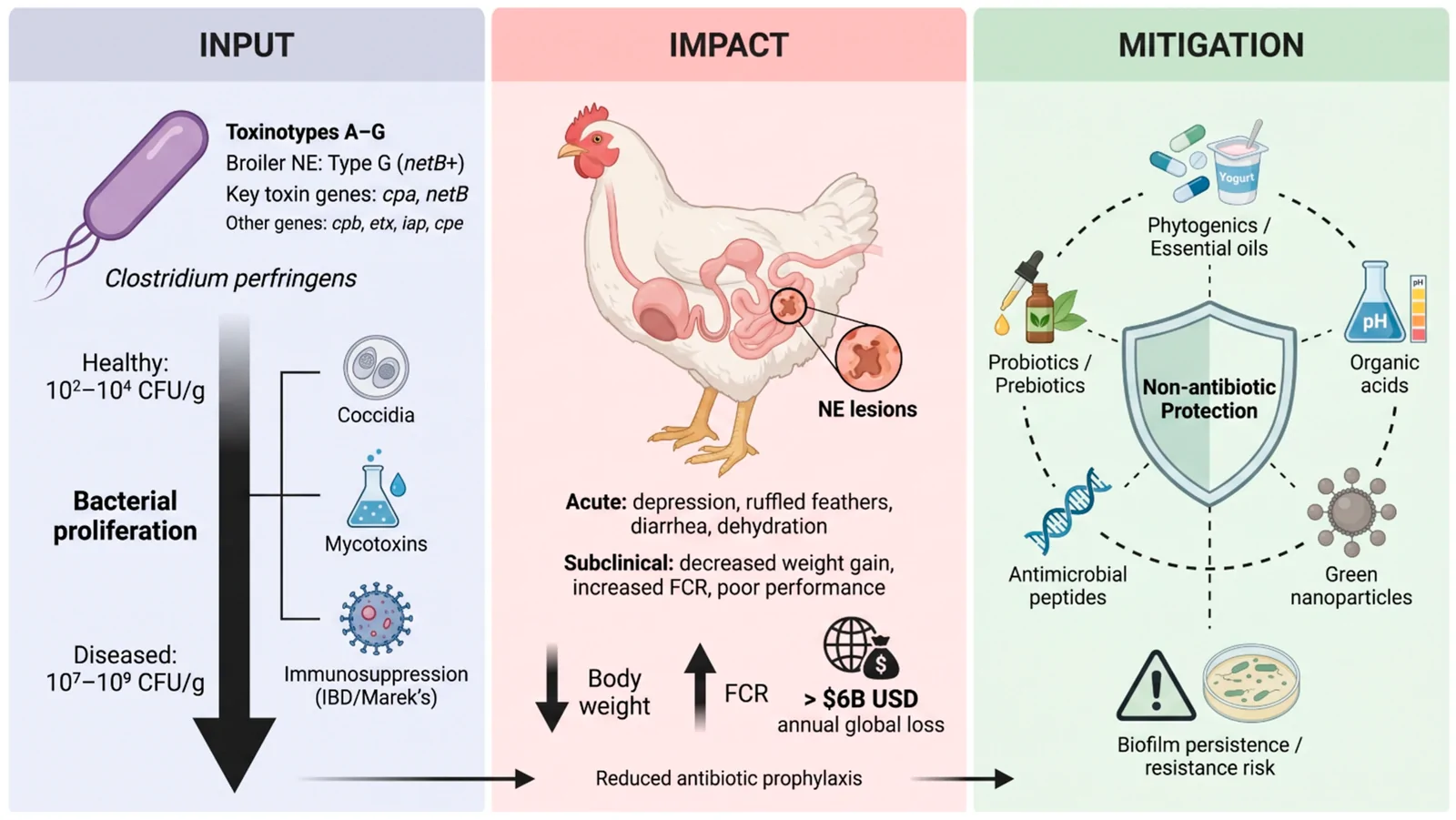
Pathogenesis, economic impact, and alternative control strategies for *C. perfringens*-induced necrotic enteritis (NE) in poultry. *C. perfringens* induces NE through extracellular toxins, leading to intestinal damage and significant annual economic losses of $6 billion USD. Excessive bacterial proliferation (up to $10^{9}$ CFU/g) is triggered by confirmed predisposing factors (Coccidia, mycotoxins, immunosuppression). Given increasing concerns regarding antimicrobial resistance and biofilm-associated persistence, integrated control strategies involving probiotics, phytogenics, and emerging nanotechnologies are being investigated as potential approaches to maintain gut health and reduce NE-associated losses.

*Clostridium perfringens* (*C. perfringens*, CP) is a Gram-positive, anaerobic, spore-forming bacterium that is a natural component of the poultry gut microbiota [1]. The presence of approximately twenty different extracellular enzymes and toxins is the basis for *C. perfringens* pathogenicity [2]. Under the expanded toxinotyping scheme, *C. perfringens* strains are classified into seven toxinotypes (A-G) according to the presence of the major toxin genes *cpa*, *cpb*, *etx*, *iap/ibp*, *cpe*, and *netB* [3,4]. α-toxin is produced by all seven types [5]. The enteritis agent alpha-toxin is characterized by its enzymatic activity and host-cell signaling features. Lately, *C. perfringens* type A has been described as the major type associated with enteritis in chickens [6,7] (Figure 1).

Necrotic enteritis (NE) is a bacterial disease caused by strains of *C. perfringens* [1], particularly types A, C, and G [8]. The disease is found in ducks, wild geese, Laysan albatrosses, mute swans, bobwhite quails, wild crows [9], and turkeys [10]. The disease is brought on by an excessive rise in the number of *C. perfringens* bacteria in the gastrointestinal tract (GIT) in combination with risk factors such as exposure to mycotoxins, diets high in non-starch polysaccharide grains, coccidia infection, and immunosuppressive viruses such as those that cause Gumboro and Marek’s disease, and chick anemia; all lead to decrease the animals’ resistance to GIT infections, causing severe NE [11,12]. The *C. perfringens* population in a healthy bird is 10^2^–10^4^ CFU/g digesta; however, during a disease, this number will rise to 10^7^–10^9^ CFU/g digesta [13] (Figure 1).

The clinical form is characterized by acute symptoms, including depression, ruffled feathers, diarrhea, dehydration, and a high feed conversion ratio (FCR). This form is severe and can result in daily mortality rates of up to 1% and a total of 10–40% deaths [14]. The most prevalent type of NE in the field is subclinical, which causes a decrease in weight gain and an increase in FCR, which ultimately results in poor performance and economic losses [14,15]. NE imposes a substantial economic burden on poultry production through mortality, reduced body-weight gain, impaired feed conversion, treatment and prevention costs, carcass losses, and delayed attainment of market weight. The global economic burden has been widely estimated at approximately USD 6 billion annually, although this figure represents an industry-level estimate rather than a harmonized global cost-of-disease analysis. Regional studies further demonstrate that the economic consequences of NE vary according to disease severity, production system, market conditions, and whether clinical or subclinical disease predominates. In particular, subclinical NE may generate substantial losses through persistent reductions in growth and efficiency despite relatively low mortality. Because directly comparable national monetary estimates remain limited, both economic metrics and experimentally quantified production losses are summarized in Table 1 to illustrate the global production burden of NE [14,15,16,17,18,19,20].

As illustrated in Table 1, geographic designation refers to the setting or scope of the cited study and should not be interpreted as a regional prevalence estimate. Monetary estimates, experimental production losses, and controlled challenge outcomes are presented separately because differences in production systems, challenge models, disease prevalence, market prices, and economic assumptions preclude direct quantitative comparison across regions.

Several reviews have addressed the pathogenesis, predisposing factors, host responses, and prevention of *C. perfringens*-associated NE, while others have focused on specific antibiotic alternatives, including probiotics, phytogenics, organic acids, and other feed additives [1,8,21,22]. More recent work has also highlighted the challenges associated with translating experimental NE-control strategies into commercial poultry production [23]. The present review extends these perspectives by integrating toxin-mediated virulence and antimicrobial resistance with the distinct biology of planktonic and biofilm-associated *C. perfringens*. Particular emphasis is placed on differentiating antimicrobial resistance from biofilm-associated tolerance and persistence and on critically evaluating non-antibiotic interventions according to the level of evidence available, including activity against planktonic cells, prevention of biofilm formation, effects on established biofilms, efficacy in experimental poultry models, and evidence from field applications. By comparatively examining probiotics, prebiotics, synbiotics, organic acids, bacteriophages, antimicrobial peptides, phytogenics, nanoparticles, and green nanotechnology within this framework, this review aims to identify current evidence gaps and the translational requirements for sustainable control of NE in poultry.

### Literature Search Strategy

This narrative review was informed by a structured literature search of PubMed/MEDLINE, Scopus, and Web of Science Core Collection from database inception through August 2026. Search terms were used individually and in Boolean combinations and included “*C. perfringens*,” “necrotic enteritis,” “poultry,” “broiler,” “toxinotype,” “NetB,” “CPB2,” “TpeL,” “alpha toxin,” “virulence,” “pathogenesis,” “quorum sensing,” “antimicrobial resistance,” “biofilm,” “persistence,” “probiotics,” “prebiotics,” “synbiotics,” “organic acids,” “phytogenics,” “essential oils,” “antimicrobial peptides,” “bacteriocins,” “bacteriophages,” “nanoparticles,” and “green nanotechnology.” Peer-reviewed original studies and relevant reviews addressing *C. perfringens* virulence, antimicrobial resistance, biofilm biology, necrotic enteritis, and non-antibiotic interventions were considered. Priority was given to recent primary studies, particularly those published from 2022 to 2026, while earlier seminal studies were retained when required to establish foundational mechanisms. Studies involving other bacterial species were included only when they provided relevant mechanistic context and were clearly distinguished from evidence directly demonstrated in *C. perfringens*. Duplicate publications, conference abstracts without sufficient data, and studies lacking direct relevance to the scope of the review were excluded. Reference lists of relevant articles were also screened to identify additional eligible studies.

## 2. Taxonomy

Taxonomy of *C. perfringens* was determined adopting the National Center for Biotechnology Information as follows: Kingdom: Bacteria, Phylum: Firmicutes, Class: Clostridia, Order: Clostridiales, Family: *Clostridiaceae*, Genus: *Clostridium*, Species: *Clostridium perfringens* or *Bacillus perfringens* or *Bacterium welchii* [24].

## 3. *C. perfringens* Toxinotypes

The pathogenic potential of *C. perfringens* is associated with a diverse repertoire of toxins and extracellular enzymes. Under the expanded toxinotyping scheme, *C. perfringens* strains are classified into seven toxinotypes (A–G) according to the presence of genes encoding the major toxins: alpha toxin (*cpa*), beta toxin (*cpb*), epsilon toxin (*etx*), iota toxin (*iap*/*ibp*), enterotoxin (*cpe*), and NetB (*netB*) [3,4].

Specifically, toxinotype A carries *cpa* without the defining major toxin genes of types B–G; toxinotype B carries *cpa*, *cpb*, and *etx*; toxinotype C carries *cpa* and *cpb*; toxinotype D carries *cpa* and *etx*; toxinotype E carries *cpa* and the iota-toxin genes; toxinotype F carries *cpa* and *cpe*; and toxinotype G carries *cpa* and *netB* [3,25,26]. Thus, enterotoxin-producing strains classified by the presence of *cpe* together with *cpa* correspond to toxinotype F, whereas toxinotype E is defined by alpha- and iota-toxin genes [3,27,28,29,30].

Under this expanded scheme, NetB-positive strains are classified as toxinotype G. Some earlier poultry studies described these isolates as type A because they were published prior to the adoption of the A-G system; historical classifications are retained when reporting those studies but clarified using current terminology [3,4].

The genes encoding α-toxin, perfringolysin O, collagenases, sialidases, hyaluronidases, and collagen adhesion protein are located on the chromosome in *C. perfringens* type A/G strains. In contrast, key NE-associated toxins, namely NetB, Beta2 toxin, and *C. perfringens* large cytotoxin (TpeL), are encoded on plasmids [31] (Table 2).

When interpreting toxin profiles, the presence of a toxin gene should be distinguished from transcription, protein production, biological activity, and demonstrated contribution to disease. PCR-based detection establishes genetic potential but does not by itself demonstrate toxin expression or causality. Accordingly, associations between *cpa*, *netB*, *cpb2*, or *tpeL* and NE should be interpreted according to the level of supporting evidence, with greater weight given to toxin-expression studies, functional assays, and infection experiments using defined mutants and complemented strains.

The pathogenic mechanism of *C. perfringens* in poultry relies heavily on a complex array of secreted protein toxins [3]. Based on their mode of action, these toxins are classified into four major functional groups: membrane-damaging enzymes, pore-forming toxins (PFTs), intracellular toxins, and hydrolytic enzymes [41,42,43,44].

### 3.1. Alpha Toxin (CPA)

Alpha toxin (CPA), encoded by the chromosomal *cpa*/*plc* gene, is a phospholipase C with well-established membrane-damaging activity [28,43]. Its complex mode of action involves binding to the target plasma membrane via the TrkA receptor, triggering a signal transduction cascade that activates protein kinase C and stimulates the arachidonic acid cascade [43,45,46]. Hydrolysis of phosphatidylcholine and sphingomyelin produces diacylglycerol and releases inflammatory mediators (including prostacyclin, thromboxane, leukotrienes, and platelet-agglutinating factors), leading to acute tissue damage and cardiovascular disruption [45,46]. CPA also acts synergistically with sialidases (which remove sialic acid to expose gangliosides) and perfringolysin O to enhance membrane toxicity and promote vascular leukostasis [22,47,48,49,50].

Historically, CPA was considered a major determinant of avian NE. However, experimental disruption of *plc* in a virulent chicken isolate did not abolish disease-producing capacity, proving that CPA is not essential for NE development in chickens [51]. Consequently, its enzymatic and tissue-damaging activities should be distinguished from an indispensable causal role, and its contribution likely depends on synergistic interactions with other virulence determinants.

### 3.2. NetB Toxin

NetB is a member of the Staphylococcal α-hemolysin family of β-pore-forming toxins (β-PFTs) [52]. It forms heptameric, hydrophilic pores with an interior diameter of approximately 26 Å that strongly prefer cations [24,53]. It promotes osmotic cell lysis by allowing Na+, Cl^−^, and Ca^2+^ to enter the cytoplasm [54,55]. Expression of *netB* is strictly regulated by the VirR/VirS two-component system [56,57] and an accessory agr-like quorum-sensing system [1,58].

NetB is the primary virulence determinant in *C. perfringens* type G infections. Early functional studies established its essential role, showing that disruption of *netB* in a virulent isolate eliminated disease-producing capacity, whereas complementation restored virulence [59]. Epidemiological surveys consistently demonstrate an enrichment of *netB*-positive and NetB-producing strains among NE-affected flocks [60,61]. However, type A or *netB*-negative strains can occasionally cause NE [62,63,64], while healthy chickens may harbor type G strains [65]. Furthermore, in vivo evaluations using ligated-loop models identified both virulent *netB*-negative isolates and low-virulence *netB*-positive strains [66]. Because non-pathogenic chicken strains are phylogenetically distinct from pathogenic avian and mammalian isolates [67], *netB* carriage alone cannot reliably define virulence. Instead, NE pathogenesis reflects the combined influence of NetB expression, strain background, regulatory networks, and host intestinal predisposing factors [66,68]. Therefore, NetB is best described as a major experimentally supported virulence determinant rather than a standalone diagnostic marker [66,68].

### 3.3. Perfringolysin O (PFO)

Perfringolysin O (PFO), encoded by *pfoA*, is a cholesterol-dependent cytolysin (CDC) produced by nearly all *C. perfringens* strains [37,69]. PFO specifically targets eukaryotic cell membranes requiring high cholesterol concentrations [70], progressing through membrane binding, cholesterol recognition, oligomerization, and insertion to form large amphipathic pores [71]. At sublethal concentrations, PFO significantly upregulates intracellular adhesion molecule 1 (ICAM-1) and CD11b/CD18 glycoprotein in endothelial cells, promoting leukocyte adhesion, local edema, and ischemia [48,49]. At high concentrations, PFO exhibits leukocytotoxic effects, disrupting endothelial integrity and causing systemic shock and multiorgan failure [49,50]. Deletion of CDC genes significantly reduces bacterial pathogenicity, highlighting CDCs as potential therapeutic targets [72].

### 3.4. Beta2 Toxin (CPB2)

Beta2 toxin (CPB2) is a plasmid-encoded 28 kDa cytolytic pore-forming toxin sharing functional sequence homology with β-toxin (CPB) [5,73,74]. First identified in a piglet with enteritis [74], *cpb2* has since been detected across multiple host species [75], encoded by either “consensus” or “atypical” gene variants [76,77]. In vitro, CPB2 induces cell rounding and lysis at concentrations >20 μg/mL via membrane disruption [78]. Although *cpb2* carriage is documented in diseased animals [79], *cpb2*-positive strains are also frequently recovered from healthy livestock and poultry [76]. In chickens, available evidence supports a potential contributory role rather than an independently established causal role in NE. Thus, detection of cpb2 should be reported as a virulence-associated genotype unless active toxin expression and functional activity are demonstrated [32,80].

### 3.5. TpeL Toxin

TpeL is a 205 kDa plasmid-encoded toxin belonging to the large clostridial glucosylating toxin family [41,81], making it the largest toxin produced by *C. perfringens* [82]. Produced during vegetative growth or sporulation [81], TpeL expression is suppressed by glucose and sucrose [40] and is sensitive to trypsin digestion [81]. TpeL binds to the host cell-surface receptor LRP1 (low-density lipoprotein receptor-related protein 1) [83] and utilizes its transmembrane and catalytic domains to deliver a glycosyltransferase enzyme into the cytoplasm, inactivating Ras signaling and triggering apoptosis [84,85].

While *tpeL* carriage alone is insufficient to cause NE [86], experimental broiler models indicate that NetB-positive/TpeL-positive isolates exhibit greater disease severity, faster infection kinetics, and higher mortality rates [82,87,88,89]. Comparing NE-producing isolates to non-pathogenic strains shows a higher co-prevalence of *cpb2*, *netB*, and *tpeL* [90]. TpeL is therefore regarded as a potent virulence-enhancing factor whose synergistic interaction with NetB remains an active area of investigation [82,89].

## 4. *C. perfringens* Virulence Factors

Several virulence factors of pathogenic *C. perfringens* result in NE progress in chickens. These virulence factors play a critical role in toxin generation, adhesion of bacteria to the mucosa (adhesins), and provision of nutrition for their fast multiplication (degradative enzymes). Several genes and genetic variants, including *netB*, *pfoA*, *cpb2*, *tpeL*, and *cna*-associated loci, have been reported at higher frequencies or associated with NE-producing isolates; however, their detection alone does not establish expression or an independent causal role in disease [16,91]. Several *C. perfringens* proteins, such as collagen adhesion protein (CNA), have been suggested to act as adhesins [92]. *C. perfringens* adherence to extracellular matrix proteins plays a significant role in the pathogenesis of NE and is highly associated with their virulence [93]. The adhesin-encoding gene *cnaA*, located in the VR-10B chromosomal region, is essential for the pathogen to adhere to gelatine and collagen types IV and V [94]. Additionally, *C. perfringens* generates an adhesive pilus that is necessary for adhering to the intestinal wall. This pilus is made up of three components, *CnaA*, *FimA*, and *FimB*, which are encoded in the VR-10B chromosomal region [94,95]. *C. perfringens* fibrinogen-binding proteins *FbpA* and *FbpB* target the extracellular matrix protein fibronectin to promote host cell interaction and subsequent colonization [16,96] (Table 3).

Sialic acid (N-acetyl neuraminic acid) is a component of the mucus glycoproteins [97]. Certain infections, such as influenza viruses, *Vibrio cholerae*, *Streptococcus pneumoniae*, and *C. perfringens*, generate sialidase enzymes that, when they release sialic acid, may provide these microbes with nutrients [98,99]. In order to produce nutrients for its growth, *C. perfringens* develops a variety of sialidases, including *NanH*, *NanI*, and *NanJ*, which improve its adherence to intestinal tissues and break the sialic acid connections at various sites [100,101]. Furthermore, intestinal damage caused by bacterial toxins induces goblet cells to secrete more mucus, which could increase the growth of harmful organisms [102]. Zinc metalloproteases, or mucinases, are additional virulence factors that are expressed by the *zmpA* and *zmpB* genes. The *zmpA* gene is found within the NELoc-1 plasmid-encoded region, which also encodes *NetB*, but the *zmpB* gene is found on a chromosome [103,104]. Proteases are primarily secreted by bacteria that live on or in mucosal surfaces. They facilitate pathogen colonization by breaking down mucin, the main glycoprotein element of mucosa [102,105]. The theory states that both proteases participate in the same catabolic process that enhances virulence, and absence of one protease is enough to stop the process and reduce virulence [103] (Table 3).

Recent studies reinforce the view that NE virulence cannot be inferred from the presence of individual toxin genes alone. Contemporary phenotypic, genomic, and transcriptomic analyses indicate considerable strain-level heterogeneity in adhesion, toxin-associated pathways, metabolic adaptation, and expression of virulence-related genes during infection. In particular, in vivo classification of diverse poultry isolates has demonstrated discordance between *netB* carriage and lesion-producing capacity, while more recent infection-associated transcriptomic analyses indicate dynamic regulation of bacterial genes during host colonization. These findings support an integrated model in which virulence emerges from strain genetic background together with environmentally responsive gene expression rather than from a single toxinotype marker [66,106] (Table 3).

## 5. Pathogenesis of *C. perfringens* in Poultry

**Figure 2:**
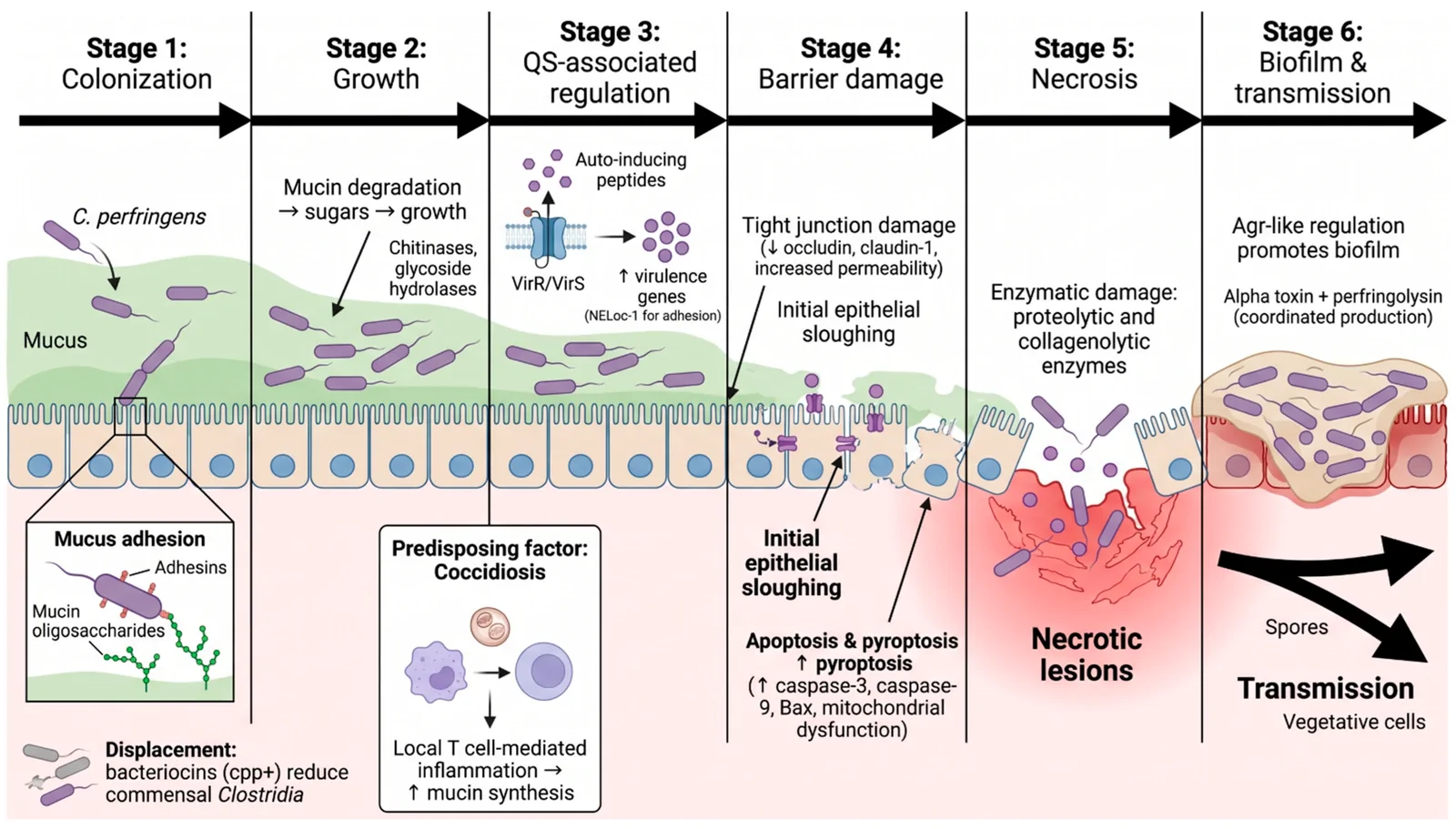
Multi-stage molecular pathogenesis of *C. perfringens* in poultry intestine. *C. perfringens* pathogenesis in poultry proceeds through six distinct stages: colonization via adhesion and bacteriocin-mediated commensal displacement; proliferation fueled by nutritional mucolysis (exacerbated by coccidiosis-induced mucin synthesis); and subsequent Agr-like quorum sensing and other regulatory networks contribute to modulation of virulence-associated gene expression. Upon establishment, the pathogen breaches the mucous layer, secreting pore-forming toxins and sloughing the epithelium. *C. perfringens* then targets the lamina propria with proteolytic enzymes, establishing a persistent Agr-regulated biofilm with alpha-toxin and perfringolysin, leading to deep tissue necrosis and subsequent environmental transmission. QS, quorum sensing.

At the molecular level, pathogenesis describes the intricate and dynamic interactions between hosts and pathogens [58,107]. Understanding the pathogenesis is important for devising control measures scientifically [108]. The six stages of the pathogenesis of a fast-growing bacterial pathogen such as *C. perfringens* are: (1) colonization of the site of preference; (2) growth and proliferation; (3) nutrition acquisition; (4) evasion of host defense mechanisms; (5) damage to host tissues; and (6) transmission [54] (Figure 2).

Mucins are secreted by intestinal epithelial cells, which provide a physical barrier that prevents infections from entering [109]. To establish and promote the progression of NE, *C. perfringens* must colonize and degrade the mucus layer [110]. Potential locations for bacterial adhesin binding are provided by oligosaccharides on the mucin glycoproteins’ core glycosylated region [111]. Bacteriocins secreted by pathogenic *C. perfringens*, such as perforins encoded by the *cpp* gene, have the ability to displace commensal *Clostridium* species that are colonizing the intestinal mucosa [112,113]. *C. perfringens* has chitinases that can break down mucins and glycoside hydrolases that can use the oligosaccharide sugars as an energy source [1]. Mucus degradation in the small intestine is probably one of *C. perfringens*’ main sources of nutrition [114]. Predisposing factors such as coccidiosis induce a local T cell-mediated inflammatory response that promotes the synthesis of mucin and mucus, both of which are necessary for *C. perfringens* growth because of its mucolytic activity [115]. This enables the bacterium to generate the QS auto-inducing peptides that initiate the VirR/VirS cascade and create localized microcolonies on the mucosal surface [116] and virulence-related genes such as *NELoc-1* involved in adhesion [58]. When the mucous layer breaks down, *C. perfringens* secretes pore-forming toxins that penetrate the epithelial cells. As *C. perfringens* spreads over the lamina propria and advances towards the intercellular junctions, it damages the basal domain of the epithelia with its proteolytic and collagenolytic enzymes. Thus, the intestinal epithelium sloughs off and becomes necrotic [117]. The development of *C. perfringens* biofilm on the exposed submucosa is facilitated by the Agr-like quorum sensing-regulated production of alpha toxin and perfringolysin [118].

Host responses are integral to NE pathogenesis and should be considered together with bacterial virulence mechanisms. Disruption of the mucus layer and epithelial junctional architecture compromises intestinal barrier integrity, increasing paracellular permeability and exposing underlying tissues to bacterial products [119]. Experimental NE models have demonstrated altered expression of genes associated with tight-junction organization, inflammatory signaling, apoptosis, and epithelial homeostasis, indicating that lesion formation reflects both direct toxin-mediated injury and active host cellular responses. Pore-forming toxins can disturb membrane integrity and ionic homeostasis and may trigger inflammatory cell-death pathways, while epithelial injury is accompanied by changes in junctional proteins, mucosal immune signaling, and tissue repair responses [120]. Recent experimental studies further demonstrate measurable increases in intestinal permeability following *C. perfringens* challenge, particularly when epithelial injury is potentiated by predisposing infections such as *Eimeria*. Several mechanistic insights have elucidated that pathogen-induced damage involves precise host epithelial remodeling, apoptotic cascades, and structural or functional dysregulation of membrane efflux complexes, particularly ATP-binding cassette (ABC) transporters [121,122,123]. Consequently, NE should be viewed as a dynamic host–pathogen process where bacterial colonization and toxin activity act together with host barrier failure, transport dysregulation, and regulated cell-death pathways [124].

Recent experimental evidence has expanded the understanding of host epithelial injury during *C. perfringens* infection. In broilers, *C. perfringens* challenge has been associated with impaired jejunal morphology, reduced expression of tight-junction components including occludin and claudin-1, and increased expression of apoptosis-associated genes such as *Caspase-3*, *Caspase-9*, and *Bax* [15]. Complementary mechanistic studies of PFO have shown disruption of epithelial junctional proteins, mitochondrial dysfunction, reactive oxygen species generation, and activation of pyroptotic responses. Collectively, these findings indicate that NE-associated tissue damage involves not only extracellular toxin-mediated membrane injury but also disruption of epithelial barrier homeostasis and regulated host-cell death pathways [120].

Contemporary NE challenge models have further demonstrated that disease phenotype reflects interactions among strain virulence, predisposing factors, intestinal microbial ecology, and host responses rather than bacterial abundance alone. Recent broiler studies comparing clinical and subclinical NE have linked disease severity with changes in jejunal microbial communities and intestinal health, reinforcing the multifactorial nature of NE pathogenesis [14,125].

## 6. Antimicrobial Resistance of *C. perfringens* Recovered from Poultry

**Figure 3:**
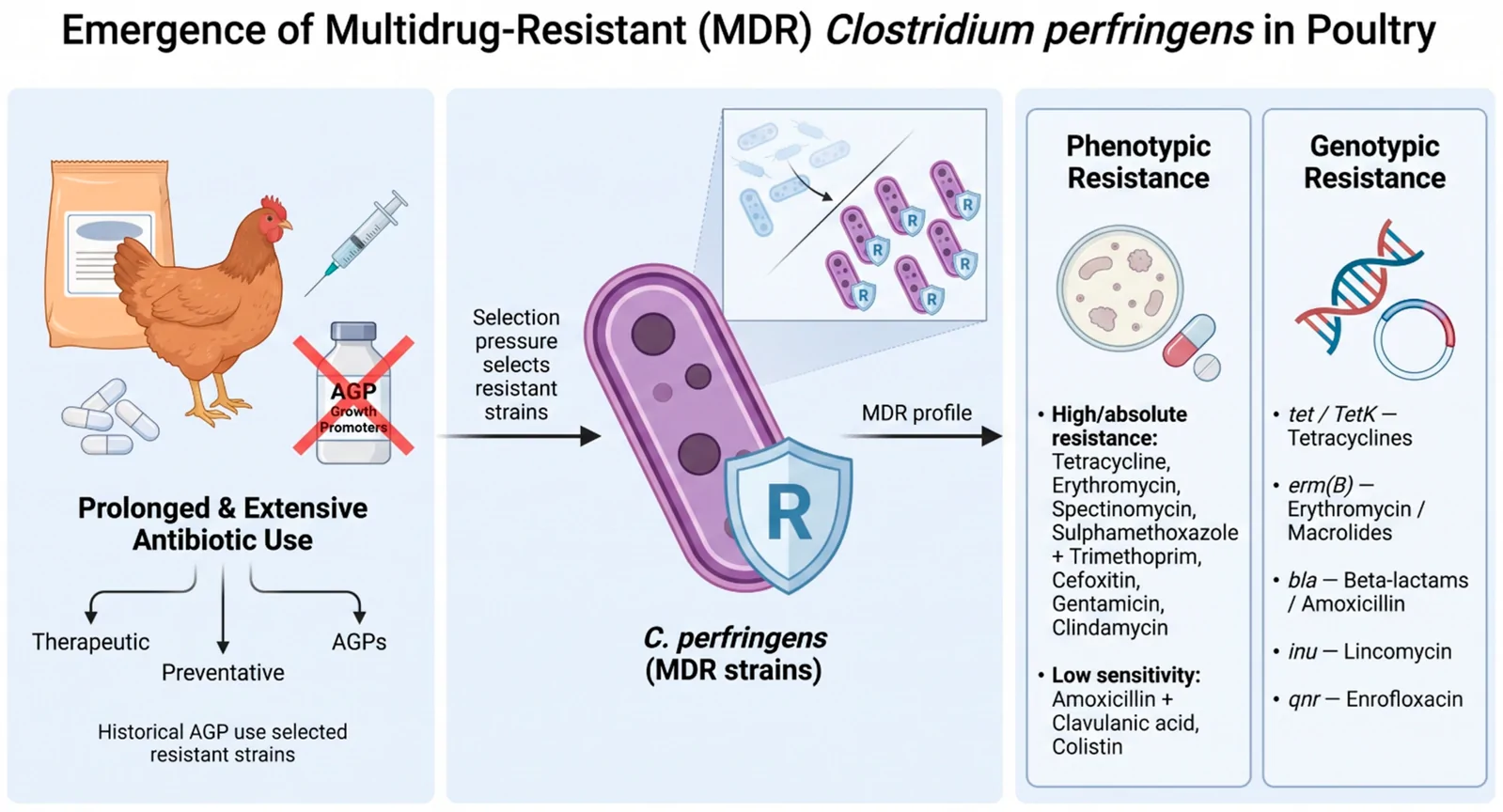
Emergence of multidrug-resistant (MDR) *C. perfringens* in poultry. Antimicrobial exposure, including therapeutic use, preventive applications, and historical antimicrobial growth promoter (AGP) usage, creates selection pressure that promotes the emergence and persistence of resistant *C. perfringens* strains. MDR isolates exhibit resistance through combined phenotypic profiles and genetic determinants, including *tet*/*TetK*, *erm*(B), *bla*, *inu*, and *qnr*, associated with resistance to tetracyclines, macrolides, beta-lactams, lincosamides, and fluoroquinolones, respectively. This highlights the increasing challenge of controlling *C. perfringens*-associated necrotic enteritis under antibiotic-restricted production systems.

Control strategies for NE involves the use of antimicrobials [126]. Antimicrobials have been used for many years to prevent NE and increase broiler production [127]. Antimicrobials are either used for therapeutic or preventative purposes in animal feed [128]. The use of antimicrobials in the poultry industry raised concerns regarding their continued usage in the antimicrobial growth promoters (AGPs) and their contribution to the development of antimicrobial resistance in bacteria [129]. Antimicrobial resistance is also a concern for *C. perfringens* infections. The prolonged and extensive use of antibiotics in poultry over the past few years has altered the bacterial environment, eliminated susceptible strains and promoted the persistence and dominance of antimicrobial-resistant bacteria [130]. For many years, antibiotics were used to promote growth; however, this practice has since been banned in a number of countries [131] (Figure 3).

Different investigations have reported multidrug resistance in *C. perfringens* isolates [132]. *C. perfringens* of foodborne illnesses has been found to be resistant to tetracycline, lincomycin, enrofloxacin, cefoxitin/ampicillin, and erythromycin through the existence of *tet*, *inu*, *qnr*, *bla*, and *erm*(B) genes, respectively, in Egypt [133]. Another study showed that absolute resistance to erythromycin, spectinomycin, and sulphamethoxazole + trimethoprim was demonstrated in 30 *C. perfringens* isolates (18 from chickens, 9 from rabbits, and 3 from calves). These isolates exhibited a high sensitivity of 90% to ampicillin + sulbactam, a moderate sensitivity of 73% and 63% to ofloxacin and cefotaxime, respectively, and 50% to each of meropenem and cefaclor. For amoxicillin + clavulanic and colistin, the isolates’ lowest sensitivity percentages were 43% and 40%, respectively. According to the Egyptian authors Eman and Sharaf [134], all examined *C. perfringens* type A isolates were multidrug resistant (MDR) showing resistance against all tested antimicrobial disks, including cefoxitin, tetracycline, gentamicin, amoxicillin + clavulanic acid, clindamycin, and sulphamethoxazole-trimethoprim (cotrimoxazole). Consequently, to validate these phenotypic findings, antimicrobial resistance genes to oxytetracycline and amoxicillin–clavulanic acid (*Bla* and *TetK*) were examined (Figure 3).

## 7. Biofilm Formation and Biofilm-Associated Antimicrobial Tolerance

Antimicrobial resistance should be distinguished from biofilm-associated tolerance and persistence. Antimicrobial resistance generally refers to a stable capacity to grow at antimicrobial concentrations that inhibit susceptible organisms and may result from acquired or intrinsic resistance determinants. In contrast, biofilm-associated tolerance reflects the enhanced survival of biofilm-embedded cells during antimicrobial exposure without necessarily involving a corresponding heritable increase in antimicrobial resistance. Persistence represents the survival of a small phenotypic subpopulation during otherwise lethal antimicrobial exposure. Restricted antimicrobial diffusion through the extracellular matrix, altered metabolic states, stress responses, and heterogeneous growth conditions may contribute to biofilm-associated tolerance but should not themselves be described as antimicrobial resistance.

In addition to its ability to produce toxins, *C. perfringens* is able to form biofilms, which increase bacterial resistance to disinfection and contributes to its continuing existence in the environment [135]. Biofilms can be described as the houses and cities of the bacterial world [136]. They are defined as “aggregates of microorganisms in which extracellular polymeric substances (EPSs) that are adhering to a surface and/or each other’s cells are frequently embedded in a self-produced matrix” [137] that undergo stages of accumulation, attachment, maturation, and dispersal [138]. Proteins, lipids, carbohydrates, and DNA make up the EPS that supports complex biofilm; each of these components increases resistance to antibiotics [139]. It was found that *C. perfringens* biofilm contained extracellular DNA, proteins, and polysaccharides for the matrix. Carbohydrates and proteins had already been identified as elements of *C. perfringens* biofilms [140,141]. A study of *C. perfringens* biofilm composition demonstrated β-1,4-linked polysaccharides and extracellular DNA as components of the biofilm matrix [140].

Biofilm-associated cells can display substantially reduced antimicrobial susceptibility compared with their planktonic counterparts [140,141], resulting in increased resistance to antimicrobial drugs and reduced effectiveness of biofilm-related infection treatment. However, the magnitude of this difference varies according to the bacterial species, antimicrobial agent, biofilm maturity, and experimental endpoint. Reports of very large fold differences originate largely from studies of other bacterial species and should not be generalized quantitatively to *C. perfringens* in the absence of directly comparable minimum inhibitory concentration (MIC), minimum biofilm inhibitory concentration (MBIC), or minimum biofilm eradication concentration (MBEC) measurements [142,143]. Bacteria can adhere to substrates and encapsulate themselves in a matrix consisting of extracellular DNA (eDNA), proteins, and EPS when they form biofilms [144,145]. Microorganisms’ surface is shielded from harm by EPS, which is the primary structural element of biofilms [146]. A linear glucosamine polymer called polysaccharide intercellular adhesin (PIA) makes up the majority of polysaccharide slime, which promotes bacterial cell attachment and biofilm formation [147]. When it comes to bacterial adhesion, extracellular DNA is essential for both the formation and maturation phases of biofilm development [148]. The combination of secreted extracellular proteins, protein subunits of cell appendages including pili and flagella, cell surface adhesions, and outer membrane vesicle proteins makes up the extracellular proteins found in the biofilm matrix. Their interactions with exopolysaccharides and nucleic acid constituents aid in the stability of the biofilm matrix, surface colonization, and preservation of the biofilm’s integrity and structure [143,149]. The biofilm layer gives the bacteria an ability to cause a variety of diseases. Thus, it is believed that bacteria that form biofilms can cause 65–80% of infections [150].

**Figure 4:**
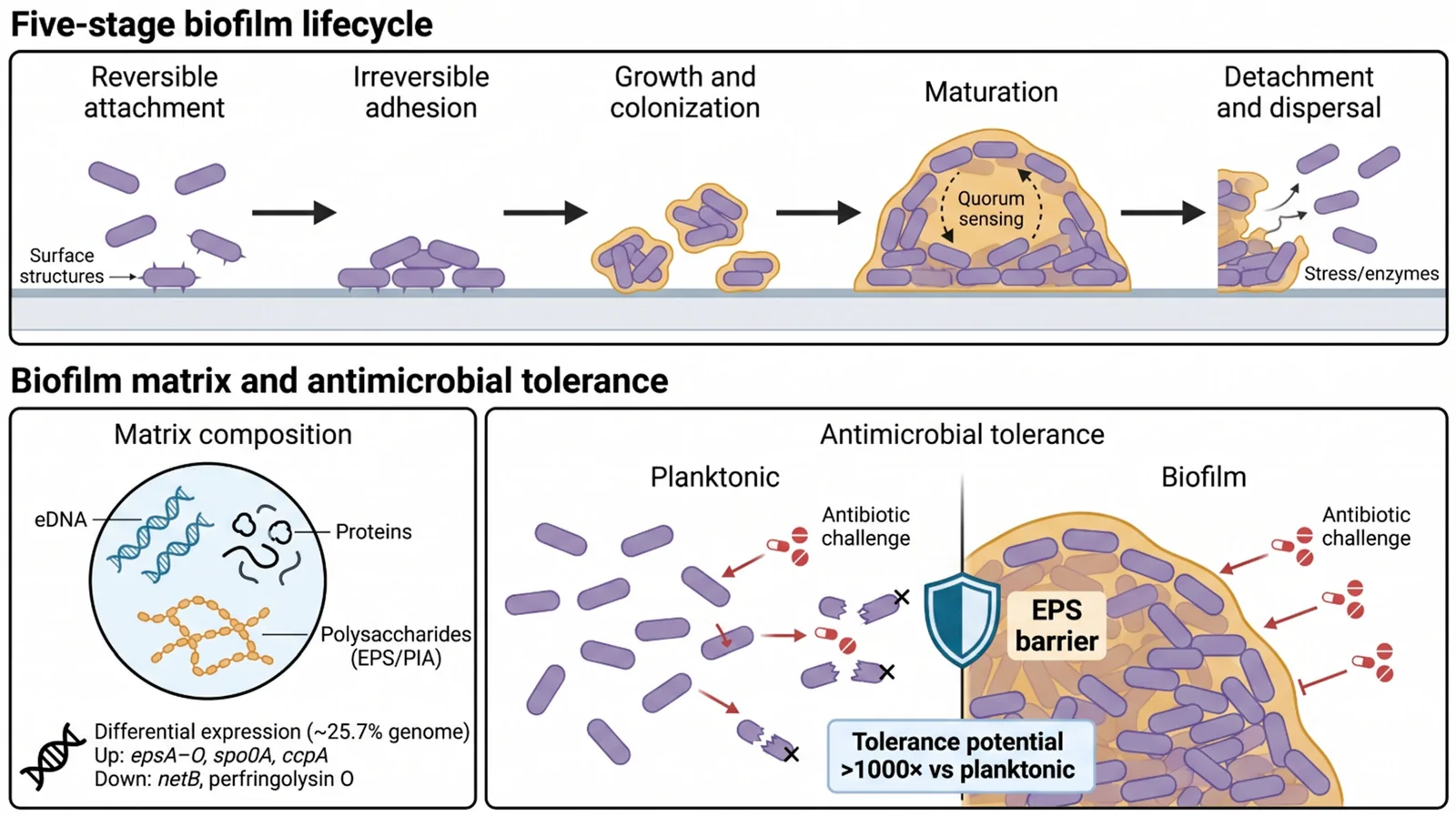
Biofilm formation by *C. perfringens* isolates. *C. perfringens* biofilms develop through a structured five-stage process; attachment, adhesion, growth, maturation, and detachment forming complex “bacterial cities” encased in a protective matrix of eDNA, proteins, and polysaccharides. This self-produced extracellular polymeric substance (EPS) acts as a physical shield contributing to environmental persistence. Biofilm-mediated infections are further regulated by a quiescent growth phase and genetic shifts in sporulation and matrix production, resulting in high rates of multidrug resistance (MDR) and extensive drug resistance (XDR) in clinical and environmental isolates. EPS, extracellular polymeric substance.

Several regulatory mechanisms play a role in regulating gene expression when *C. perfringens* forms biofilms. A total of 25.7% of the genes in *C. perfringens*, including those involved in EPS production (*epsA-O*, *tasA*, *tapA*, *bslA*, *sipW*, and *calY*), sporulation (*sigG*, *sleC*, *soj*, *spoOA*, *spoVB*, *spoVD*, and *spoIIE*), motility (*ccpA*), toxin production (*virX*, *luxS*, *codY*, and *ctrAB*), and the stress response (*hrcA*, *lexA*, and *recA*) show differential expression between biofilms and planktonic cells [151]. The downregulation of virulence genes encoding alpha-clostripain, collagenase, netB toxin, perfringolysin O, and phospholipase C indicates that the biofilm induces a quiescent growth phase and that the toxin proteins may not be directly involved in the creation of the biofilm [152]. It was proved that the type IV pilus and the catabolite regulatory protein CcpA are essential for *C. perfringens* to generate maximal biofilms [141,153]. However, hemolysins (such as perfringolysin O and alpha toxin) were found in a compound with eDNA and were increased in the biofilm, suggesting a possible increase in its pathogenicity [118]. The expression of toxin genes is probably downregulated during *C. perfringens* biofilm formation at optimal growth temperatures, but at lower temperatures, toxin genes might be involved in the synthesis of extracellular matrix to strengthen the bacterium’s adaptability to the altered environment [153] (Figure 4).

In Egypt, genotyping of thirty *C. perfringens* isolates (20 diseased and 10 from chicken carcasses) revealed genotype A (*cpa+*). The virulence genes *cpe*, *cpb2*, *netB*, and *tpeL* were detected with percentages of 6.7%, 56.7%, 56.7%, and 36.7%, respectively. Eight isolates (26.7%) were XDR, one isolate (3.3%) was PDR, and twenty-one isolates (70%) were MDR. A multiple antibiotic resistance index of 0.7 was averaged. Of the *C. perfringens* isolates, 63.3% formed biofilms, which were divided into three categories: weak (36.7%), moderate (16.7%), and strong (10%). *C. perfringens* biofilms were significantly reduced by sodium hypochlorite [154]. In another Egyptian study, fifty *C. perfringens* isolates from various sources were tested for antibiotic susceptibility, and the results showed that 82–94% of the isolates were resistant to penicillin, cefotaxime, cefoxitin, ceftriaxone, clindamycin, and chloramphenicol. On the other hand, 86% of the isolates showed metronidazole susceptibility and all of them were susceptible to vancomycin. In addition, 74% of the studied isolates were ampicillin, amoxicillin, and ampicillin-sulbactam sensitive. Ninety-two percent of the isolates (46/50) showed multiple drug resistance, and the multiple antibiotic resistance (MAR) index had an average of 0.63 and ranged from 0.28 to 0.9. All *C. perfringens* isolates were examined for biofilm production; of these, 14 (28%) were considered strong biofilm producers, which were categorized as XDR with a MAR index of ≥0.7, 60% (30/50) were moderate biofilm producers, which considered MDR with MAR indices ranging from 0.2 to 0.6 [155].

More recent studies have extended *C. perfringens*-specific biofilm research beyond descriptive characterization toward targeted biofilm control. For example, contemporary poultry-associated studies have demonstrated isolate-dependent biofilm phenotypes and evaluated thymol as a direct inhibitor of *C. perfringens* biofilm formation. These findings support the need to evaluate candidate interventions using organism-specific biofilm models rather than extrapolating antibiofilm mechanisms from unrelated bacterial species [156].

As shown in Figure 4, the five steps in formation and development of biofilm are as follows: (1) reversible attachment, (2) irreversible adhesion, (3) growth, (4) biofilm maturation, and (5) detachment [157,158]. In the first stage, free-floating bacteria attached firmly to the substrate surface. The production of extracellular polymeric materials, proteins, and other biomolecules by bacteria during the colonization stage of biofilm development increases adhesion strength. Microorganisms in the biofilm use QS to interact with one another as they mature. The microorganisms separate from the biofilm and attach to the surrounding media during the last stage of biofilm formation [159]. The detachment is caused by various factors, and these factors are external disturbance, endogenous enzyme depredation, release of EPS, surface binding proteins and starvation [160,161,162]. Fimbriae, flagella, and capsules are produced by bacteria in biofilms to help them adhere firmly to surfaces [149].

## 8. Mechanisms Contributing to Biofilm-Associated Antimicrobial Tolerance

Available evidence indicates that *C. perfringens* biofilms contain extracellular DNA, proteins, and polysaccharide components, and that type IV pili and regulatory factors such as CcpA contribute to biofilm development. These mechanisms are supported by direct studies of *C. perfringens* [140,141,142,143,144,145,146,147,148,149,150,151,152,153]. In contrast, several mechanisms frequently described in other bacterial biofilms, including specific exopolysaccharides, matrix-associated antibiotic-inactivating enzymes, and particular efflux or cation-response systems, have not been adequately demonstrated in *C. perfringens*. Such mechanisms should therefore be considered general conceptual models rather than established components of *C. perfringens* biofilm physiology.

For several decades, the poultry industry has regularly utilized subtherapeutic dosages of antibiotics as AGPs in broiler chickens to improve feed efficiency (FE) and decrease disease incidence [163]. However, improper, and excessive use of antibiotics in livestock farming has resulted in the emergence of bacteria resistant to these drugs, which poses a serious risk to both human and animal health [164]. As a result, in animal agriculture, the use of AGPs was banned by the European Union in 2006 [165]. Consequently, there has been a growing interest in finding viable replacements for antibiotics as growth promoters in broiler chickens [166].

## 9. New Approaches Against *C. perfringens* Isolated from Poultry

**Figure 5:**
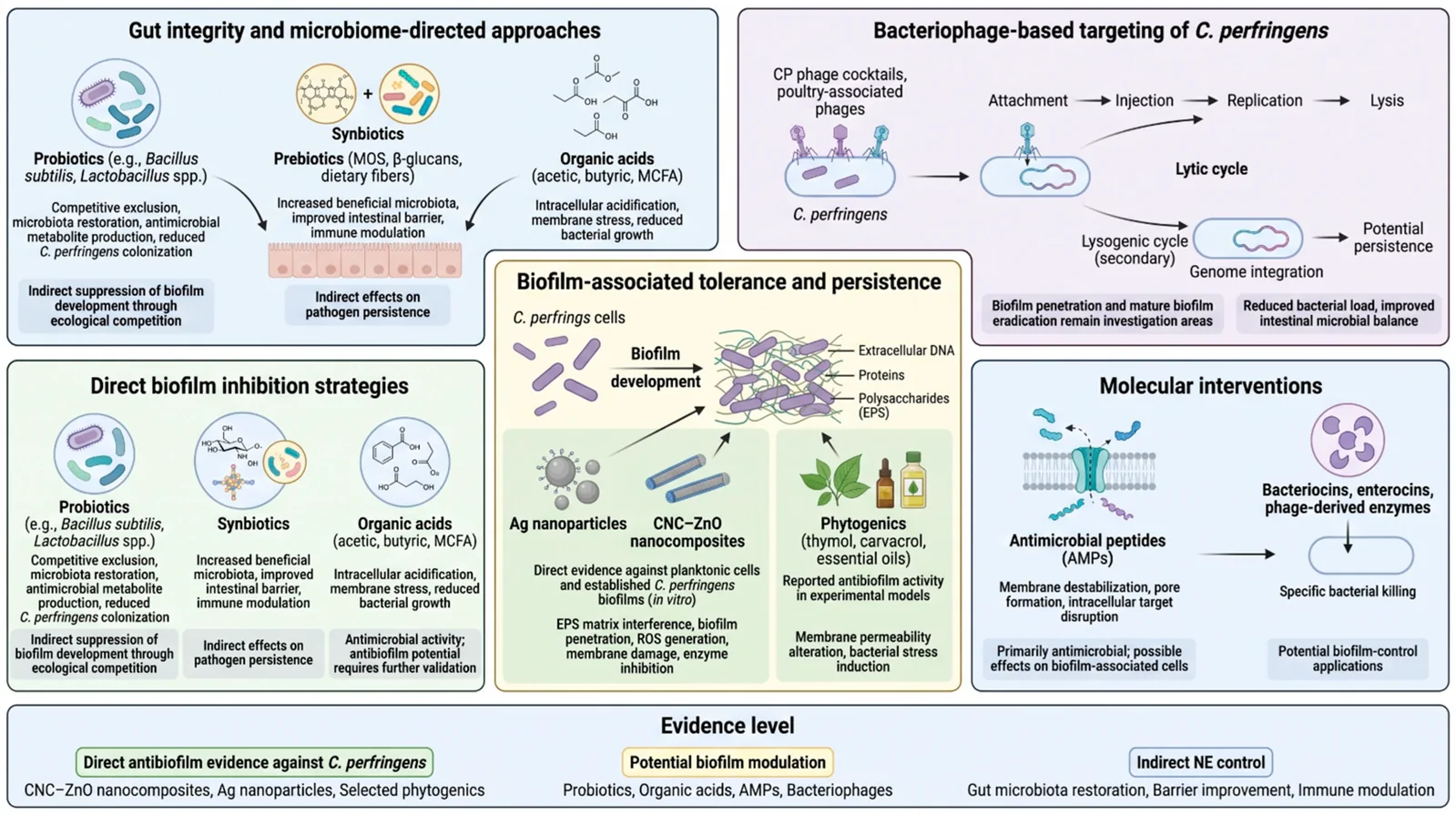
Integrated non-antibiotic therapeutic strategies for the management of *C. perfringens* and necrotic enteritis in poultry. The management of *C. perfringens* in poultry utilizes a multi-pronged approach featuring probiotics, prebiotics, and organic acids that work collectively to restore gut microbiota, enhance intestinal morphology, and disrupt bacterial cellular physiology. Innovative biological and molecular interventions, such as bacteriophage cocktails and antimicrobial peptides, provide targeted bactericidal activity and non-selective membrane destabilization to neutralize virulent toxinotypes. Furthermore, green-synthesized nanoparticles and phytogenic compounds offer sustainable alternatives that inhibit biofilm formation and downregulate virulence genes, ultimately improving feed efficiency and reducing necrotic enteritis lesions. Ag NP, silver nanoparticles; AMPs, antimicrobial peptides; CNC-ZnO, cellulose nanocrystal–zinc oxide; CPQ, *C. perfringens* phage cocktail; DHFR/DHPS, dihydrofolate reductase/dihydropteroate synthase; MCFAs, medium-chain fatty acids; MDR, multidrug-resistant; NE, necrotic enteritis; NPs, nanoparticles; NTZ, nitazoxanide; PDR, pandrug-resistant; ROS, reactive oxygen species; ZnO NP, zinc oxide nanoparticle.

The evidence supporting non-antibiotic interventions differs substantially according to the experimental endpoint. In this review, efficacy is therefore distinguished among: (i) inhibition or killing of planktonic *C. perfringens*; (ii) prevention of initial biofilm formation; (iii) disruption or eradication of established mature biofilms; (iv) reduction in NE severity in controlled poultry challenge models; and (v) effectiveness under commercial or field conditions. These endpoints should not be considered interchangeable, because in vitro antimicrobial or antibiofilm activity does not necessarily predict therapeutic efficacy or production benefits in vivo (Figure 5).

### 9.1. Probiotics

Live cultures of benign bacteria or yeast species, known as probiotics, are being used more frequently in poultry feeds in place of AGPs [167]. Probiotics from the genera *Bacillus*, *Lactobacillus*, *Enterococcus*, and others have been tested against *C. perfringens* both in vivo and in vitro [168]. Through competitive exclusion, they maintain a regular gut microbiota, which benefits health. Additional probiotics exhibit antagonistic properties towards infections, modify microbial metabolism, and generate advantageous metabolites, thus enhancing feed intake, digestion, and immune system performance [169,170]. By using a competitive exclusion mechanism, probiotics can also prevent harmful bacteria like *Salmonella* spp. and *C. perfringens* from growing and colonizing in the intestines [21]. Since probiotics have been shown to have a preventive effect on clostridial disease, it has been established that probiotic cultures possess significant antibacterial potential to be utilized as biological agents to prevent *C. perfringens* infections [171]. It has been demonstrated that adding probiotic *Bacillus subtilis* to broiler diets reduces the amounts of harmful bacteria in the GIT, enhances intestinal integrity and nutrient retention, and improves feed conversion [172,173,174]. A previous research on broilers with NE showed that *B. subtilis* increased the abundances of beneficial commensal microbes and associated compounds, which in turn improved growth performance and reduced intestinal inflammation [175,176]. Economic savings of $0.018 USD/kg of body weight were also possible with probiotics when *B. subtilis* was incorporated into a diet with a two percent reduction in metabolizable energy [177] (Figure 5).

While general probiotic mechanisms include restoration of the perturbed microbiota, production of antimicrobial molecules, competitive exclusion of pathogens from colonization, and host immune modulation, the precise mechanisms by which probiotics control *C. perfringens*-induced NE depend on a number of factors, including the species and strain of the probiotic agent, age and type of the bird, host immune status, and importantly, the severity of NE [21]. Probiotics and their mechanism of action to prevent NE in poultry are presented in Table 4.

### 9.2. Prebiotics

Prebiotics are more advantageous than probiotics because, in contrast to probiotics, they are designed to specifically support beneficial bacteria that are already present in the intestines [204].

Prebiotics include fiber, polyphenols, polyunsaturated fatty acids, and conjugated linoleic acid; they are health-promoting substrates for microorganisms [205,206]. Prebiotics are not able to be broken down and absorbed by fowl when given as a nutritional supplement. But they can also be very important for intestinal microecology, for encouraging the growth of specific bacteria, and for boosting nutrient absorption and utilization [207,208]. They also reduce the severity and duration of the lesions caused by *C. perfringens* in the intestine especially in jejunum and duodenum [209] (Figure 5).

A potential substitute for antibiotics has been investigated in *Saccharomyces cerevisiae*’s yeast cell wall (YCW) [210,211]. YCW extracts, which include mannan oligosaccharides (MOSs), mannan proteins, β (1,3)- and β (1,6)-glucans, chitin, and glycophospholipid surface proteins linked to the plasma membrane, have found numerous uses [212,213]. Therefore, feeding broiler chickens with dietary supplements comprising YCW extracts containing varying amounts of MOS and β-glucans may enhance gut morphology and growth performance, encourage the development of immune organs, increase the secretion of gut immunoglobulin, and hinder pathogenic bacteria from colonizing the gut [212]. It has been reported that dietary supplementation with MOS improves gut morphology, resulting in longer villi, shorter crypts, and more goblet cells [214]. MOS influenced immunological and metabolic pathways in the gut of infected chicks (*Salmonella* Typhimurium and *C. perfringens*) in a way that decreased colonization [215,216]. Dietary supplementation with β-glucans increased villi height in the gut and antibody levels against *C. perfringens* [217]. Supplementing chicks with β-glucans also enhanced the expression of genes related to innate and adaptive immunity, like β-defensins and cathelicidins, in their guts when they were challenged with NE [218,219,220].

### 9.3. Synbiotics

A probiotic and prebiotic combination is known as synbiotics; they work in concert to improve the colonization and survival of good bacteria in the digestive system [221,222]. Synbiotics help the host by producing antimicrobial chemicals, competitive exclusion, antibodies that modulate the immunological response, immune cells that enhance phagocytosis, natural killer cell activity, and anti-inflammatory cytokines [223]. In response to NE exposure, synbiotic supplementation enhances production performance, lowers lesion score, and stimulates a positive humoral and cell-mediated immune response [224]. The potential of a synbiotic feed additive including *E. faecium*, *S. cerevisiae*, and *Bacillus* species to prevent NE in broilers challenged with *C. perfringens*, a bacitracin-resistant pathogen, was assessed [225] (Figure 5).

### 9.4. Organic Acids

**Figure 6:**
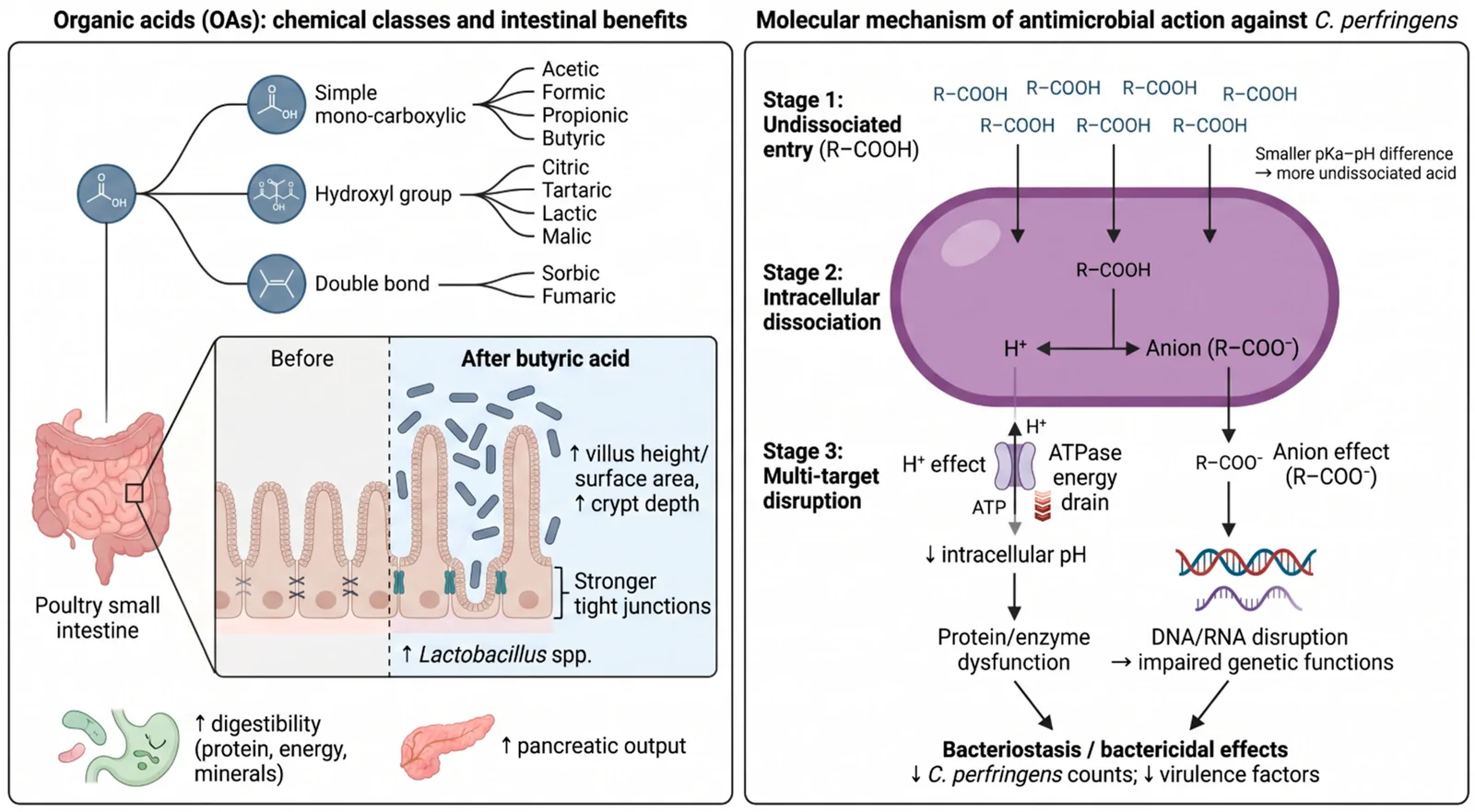
Mechanistic basis of organic acids (OAs) as multi-target antimicrobial agents in poultry. Organic acids (OAs) improve the broiler health and intestinal integrity by promoting gastrointestinal cell proliferation, notably increasing villus height and crypt depth, while serving as potent non-antibiotic antimicrobial agents. Mechanistically, undissociated OAs penetrate the bacterial cell wall and dissociate intracellularly, causing a critical drop in internal pH that denatures proteins and exhausts cellular energy (ATP) via proton-pump activity. Simultaneously, the anionic fraction of the acid disrupts bacterial replication and translation by interacting with nucleic acids, effectively downregulating virulence factors and neutralizing pathogens like *C. perfringens*.

Organic acids (OAs) are carboxylic acids, which include fatty acids and have the chemical structure R-COOH along with certain other properties. Three classes can be distinguished from them: (a) simple mono-carboxylic acids, such as acetic, formic, propionic, and butyric acids; (b) carboxylic acids with hydroxyl groups, such as citric, tartaric, lactic, and malic acids; and (c) carboxylic acids with double bonds, such as sorbic and fumaric acids [226]. Specific lipid groups such as polyunsaturated, unsaturated, or saturated fats or fats classified as short-, medium-, or long-chain fatty acids based on the length of their fatty acid chains are the most often studied lipid groups in research. Short- and medium-chain fatty acids (SCFAs and MCFAs, respectively) have been shown to improve broiler health, production, feed digestibility, and reduce body and muscle fat [227,228] (Figure 6).

Because organic acids and their salts are usually regarded as safe, the European Union permitted their use in the production of chickens [229]. The benefits of organic acids are varied and include antibacterial properties, the promotion of intestinal integrity or gut development, enhanced nutritional digestibility, and an increased population of beneficial microbiomes (*Lactobacillus* spp.) [230]. Organic acids have antimicrobial properties as well as improving protein, energy, and mineral utilization in addition to increasing pancreatic output. Butyric acid exerted its highest effect on gastrointestinal cell proliferation, resulting in increases in villus height, surface area, and crypt depth [231]. Through increased intestinal barrier function, intestinal microbiota, and volatile short-chain fatty acid (SCFA) synthesis, dietary supplementation of various mixes of OA and/or phenolic compounds can mitigate the deleterious effects of NE challenge on intestinal health. When in-feed AGP is limited to use in chicken production, these results collectively show how OA mixes may be able to lessen the adverse effects of NE [232]. One possible mechanism of action for organic acids is non-dissociation, in which they penetrate the bacterial cell wall and modify the normal physiology of the bacteria, resulting in an internal pH change and the dissociation of H+ and anions, which consumes energy and may even kill the bacteria [8]. They also activate the intestinal mucosa, alter the microbiota, and have immunomodulatory effects. The type, dosage, and basis of the organic acid product determine the effects on broilers [233]. Lauric, capric-caprylic, and caproic acids are linked to reduced stool *C. perfringens* counts and enhanced gut histomorphology [234].

#### Antimicrobial Activity of Short-Chain Fatty Acids (SCFAs) or Medium-Chain Fatty Acids (MCFAs)

As mentioned above, in addition, the anionic component of the acid interacts directly with the free nucleic acids in the cytoplasm of the bacterium, impacting translation, transduction, and replication processes. This is because the bacterium lacks a nuclear membrane. Microorganisms’ pathogenic potential and the expression of resistance mechanisms would be negatively impacted by bacterial pathogenesis, which is dependent on the expression of virulence factors encoded at the DNA and which rely on transduction and translation mechanisms [235]. On the other hand, the acid’s cationic fraction (H+) lowers the bacterium’s internal pH, denaturing proteins and influencing enzyme activity. Because the pH affects the catabolic activity of the enzymes, a reduction in pH modifies the isoelectric point required for catabolic activation and lowers bacterial metabolism, resulting in bacteriostasis [236]. The bacterium uses membrane protein complexes (ATPases) to try and remove hydrogen ions to reverse the pH drop, but this requires a large energy expenditure, thus starting a bactericidal, biochemical, and negative physical cycle [235]. Since MCFAs have a greater pKa value than other organic acids, they are better at regulating microbial growth than they are at lowering pH [237,238]. Furthermore, a higher pKa value relative to the pH in the colon, small intestine, ventriculus, or gizzard will cause the balance to shift further towards the undissociated form, which will have a greater antibacterial impact [239]. The mechanism of action of organic acids and SCFAs as antimicrobials against *C. perfringens* is shown in Figure 6.

### 9.5. Bacteriophages

Bacteriophages infect and replicate inside a bacterial host and serve as natural biocontrol agents [240]. Bacteriophages and bacteria have a long-standing synbiotic and competitive relationship. In contrast to other antibiotic alternatives, bacteriophages are a unique class of living organisms that multiply by lysing the bacterium and automatically exploring the host’s surface-specific receptors [241,242].

Bacteriophages have four structural forms that make up their genetic material: ssDNA, dsDNA, ssRNA, and dsRNA. These forms are very adaptive to their surroundings. Bacteriophages select their genetic information; replication mechanisms based on several life cycle modes (lytic, lysogenic, and chronic) after they have entered the host bacterial strain [242,243]. Numerous studies have demonstrated the potential benefits of dietary bacteriophage supplementation in regulating intestinal microbial composition, improving mucosal immunity and the physical barrier function of the intestinal tract, and supporting intestinal microecological stability and normal animal body development [243]. Because of their comparatively high bactericidal activity, bacteriophages (phages) can damage bacterial host cells [244]. From chicken meat, phages targeting *C. perfringens* were successfully identified and given morphological and genetic characterizations. The significant potential of a 4-lytic phage cocktail (CPQ3, CPQ7, CPQ8, and CPQ10) as a natural biocontrol agent against *C. perfringens* in food and in vitro was documented [245].

As a potential substitute for the control and prevention of *C. perfringens* in chickens, a phage called HN02 has been demonstrated to be effective in reducing the pathogen’s contamination [246]. Thanki et al. [247] reported that out of the 32 recovered phages, 19 lysed 87–92% of the poultry strain collection worldwide (*n* = 97). In liquid culture, the pathogenicity of each of these individual phages, as well as 32 distinct phage combinations, was measured at various dosages. Next, in order to replicate an industry-used successful poultry model, a multi-strain infection model was created for *C. perfringens* larvae. Using the larva model, the effectiveness of 16/32 phage combinations was evaluated. This led us to conclude that, when given prior to bacterial challenge, the phage cocktail which included the phages CPLM2, CPLM15, and CPLS41 was the most successful at decreasing *C. perfringens* colonization in infected larvae. These findings imply that phages do provide a great deal of promise for treating and preventing *C. perfringens* infections in chickens. Mohd Shaufi et al. [248] reported that adding probiotics and phage cocktail to poultry diets has major favorable effects. In particular, the addition of 1 g/kg of probiotics and 1 g/kg of phage cocktail considerably enhanced the growth performance and decreased *C. perfringens* populations. A study in Egypt reported that NE in broilers could be effectively treated in experiments using the isolated podovirus *C. perfringens* phage [249].

### 9.6. Nanoparticles as Antimicrobial Agents Against C. perfringens

It has been demonstrated that nano-minerals or nanoparticles (NPs) improve growth, egg quality and production, immunological modulation, and antioxidant status for poultry. They also help to save production costs by lowering the need for additional mineral doses and increasing the feed conversion ratio. Additionally, they appear to be less hazardous than traditional mineral sources [250]. Consequently, nano-minerals may be used as mineral supplements as well as antimicrobial feed additives. In addition to nano-essential minerals, nanomaterials have the potential to modify the gut microbiome by directly preventing microbial development or by changing the way microbial metabolism occurs in the gut [251]. Eman and Sharaf [134] reported that nitazoxanide (NTZ) treats antibiotic-resistant *C. perfringens*-infected poultry without having a deleterious biochemical impact on liver or kidney functioning. Torky et al. [252] reported that Ag NPs have potent antibacterial action against MDR strains of *C. perfringens*. This study is the first, as far as the authors are aware, that examined the possible alternate antibacterial role of Ag NPs for the management of MDR *C. perfringens* in Egypt. Another study in Egypt demonstrated that 100 μg/mL of Ag NPs could prevent the production of biofilms in *C. perfringens* isolates recovered from humans, camels, and chickens. For the pigeon isolate, no biofilm formation was seen at a concentration of 75 μg/mL [155].

### 9.7. Antimicrobial Peptides as Antimicrobial Agents Against C. perfringens

Antimicrobial peptides (AMPs) are small protein molecules that are present in nearly all living things [253,254,255]. They have developed as a means of protecting the host against microbes and are crucial to innate immunity [256]. AMPs provide a number of benefits, including the capacity to function in multiple ways, ease of degradation in the environment, decreased accumulation, enhanced host immunity, neutralization of numerous microorganisms’ activities, and apparent low resistance frequency [254,257]. AMPs present broad-spectrum antimicrobial activity against bacteria, fungi, protozoa and viruses [258]. Because AMPs target non-selectively, they are more effective in eliminating MDR pathogens [258]. It is more difficult for bacteria to evolve resistance to the electrostatic interactions between AMPs and anionic cell membrane components than to develop resistance by modifying specific membrane targets. When it comes to their antibacterial, antifungal, and even antiviral characteristics, AMPs like lactoferrin are more adaptable (or non-specific) than antibiotics [259,260].

Electroporation, non-lytic membrane depolarization, membrane destabilization, pore formation, membrane thinning or thickening, and oxidized lipid targeting are some of the methods by which the majority of AMPs disturb the bacterial membrane [261]. However, certain AMPs can also interact with intracellular targets, interfering with bacterial metabolic turnover and limiting the synthesis of proteins, acids, and cell walls [262]. Because of their bactericidal or bacteriostatic properties, AMPs can be utilized to promote the broilers’ growth and to treat or prevent bacterial infections. In the avian gut, AMPs have been demonstrated to dramatically lower the number of pathogenic bacteria while simultaneously boosting the number of beneficial microbes and modifying the intestinal microbiota [254,263]. García-Vela et al. [264] reported that because of their potent activity against *C. perfringens* and other pathogens, synthetic enterocins, either alone or in combination, represent a promising alternative to antibiotics in the poultry industry. *Pediococcus pentaceus* FBB61 produces the bacteriocin pediocin A, which has shown antibacterial activity against *C. perfringens* type A [265]. In a different investigation, drinking water containing the peptide sublancin, which is generated by *Bacillus subtilis* 168, was given to chickens. The authors showed that this AMP might be employed as a possible antibacterial agent to manage *C. perfringens*-caused NE without altering the *Lactobacillus* community [266]. The food industry currently uses nisin, a bacteriocin generated by *Lactococcus lactis*, as an additive (GRAS, E234 in the EU) because of its ability to suppress potential bacterial infections [267]. Nevertheless, some studies have investigated its use in poultry. Numerous studies have shown that adding nisin to the diet alters the gut microbiota of the animal, lowering the potential of infections such as *C. perfringens* developing in the ileum [268]. García-Vela et al. [264] reported that a panel of twenty *C. perfringens* isolates was used to evaluate the inhibitory activity of a chemically synthesized set of enterocins from various subgroups of class II bacteriocins, tested individually and in combination, including class IIa enterocins A, P, and SEK4; the class IId leaderless enterocin L50; and the unsubgrouped class II enterocin B. The results showed that synthetic analogs of L50A and L50B were the most effective against *C. perfringens*.

### 9.8. Phytogenics as Antimicrobial Agents Against C. perfringens

Phytobiotic feed additives can be defined as plants or herbs that are added to animal feed to enhance animal performance. Herbs (non-woody, non-persistent plants), spices (boldly flavored), and essential oils (EOs) are examples of phytobiotics [269]. Phytogenic feed additives, or PFAs, have been shown in numerous studies to provide a range of benefits, including enhanced feed palatability, intestinal development, and health, as well as antibacterial, antiviral, antioxidant, antihelminthic, anti-inflammatory, and anti-stress responses [270]. Since the use of antibiotics in the feed industry is prohibited, they are mostly used as alternative AGPs in the production of nonruminant livestock (swine and poultry) [271]. Phytogenic compound (PC) can be utilized as extracts (crude or concentrated) or in solid, dried, and grinding forms [272]. Three primary groups of biologically active PCs have been identified: (1) phenolic compounds (flavonoids, phenolic acids, lignin, lignans, coumarins, stilbenes, and tannins); (2) terpenoids (plant volatiles, sterols, carotenoids, saponins, and glycosides); and (3) nitrogen-containing substances (alkaloids, glucosinolates, and cyanogenic glycosides) [273]. Considering the multitude of aromatic plants, herbs, and individual chemicals, their various modes of action are characterized by diversity and depend on their specific combination, concentration, and chemical composition.

The antibacterial activity of PCs is one of their remarkable qualities. Research on the antimicrobial qualities of PCs made from herbs and spices is quite comprehensive [274]. Thymol, carvacrol, eugenol, and cinnamon aldehyde are examples of plant-derived EOs that are frequently used as in-feed antibiotic alternatives to enhance the performance of livestock and poultry, boost intestinal health, and control chicken intestinal pathogens such as *C. perfringens* [275,276,277], because of their potent antioxidant, bactericidal, and antibacterial properties as well as their capacity to increase the secretion of intestinal digesting enzymes without leaving behind harmful residues [278]. When compared to antibiotics, EOs showed stronger antibacterial activity against *C. perfringens* in the in vitro investigation. When compared to untreated birds, the in vivo study revealed that birds treated with EOs had significantly lower bacterial counts, intestinal lesions, and improved performance [279]. Another study showed that supplementation with *Scutellaria baicalensis* and *Lonicerae Flos* (SL) extract could improve intestinal barrier function and histomorphology, hence increasing the growth performance of challenged birds and effectively mitigating the adverse impacts of *C. perfringens* challenge [280]. Furthermore, the research has shown that a combination of hydrophilic organic acids and hydrophobic EOs is more effective than either one alone at inhibiting harmful microbes [281,282]. It demonstrates beneficial effects on intestinal microbiota and immune regulation in chickens, strengthens intestinal barrier function, and eventually improves feed efficiency and growth performance in poultry [283], in addition to managing subclinical infections in chickens brought on by enteric pathogens such as *Salmonella*, *E. coli*, *C. perfringens*, *C. jejuni*, and *Coccidia* infections [284].

The potential synergistic effect of hydrophobic EOs and plant-derived organic acids could lie in their ability to impair the integrity of bacterial cell membranes, thereby facilitating the entry of hydrophilic organic acids into bacterial cells and their subsequent achievement of antibacterial and bactericidal effects [285,286].

### 9.9. Green Nanotechnology

Green synthesis, which uses the principles of green chemistry to create new nanomaterials, has the potential to drastically change industrial-scale nanomanufacturing processes [287]. Greener nanomaterials may play an increasingly important role in nanomedicine in the years to come as innovative drug delivery carriers and for other uses. Because green synthesis operates at room temperature and is easily scalable, it does not require costly equipment and is comparatively faster [288]. The process of creating nanoparticles with biomedical uses by utilizing microbes and plants is known as biosynthesis. This method is safe, green, economical, biocompatible, and environmentally friendly [289]. Green synthesis involves the use of bacteria, fungi, algae, plants, and other microorganisms. They enable the large-scale, impurity-free synthesis of ZnO NPs [290]. NPs synthesized using a biomimetic technique exhibit enhanced catalytic activity while reducing the need for costly and toxic chemicals [291]. The most favored source of NP synthesis is plants since they produce stable NPs with a wide range of sizes and shapes on large scales [292]. Wet chemically produced NPs have unique qualities, such as increased antibacterial efficacy of up to 99.9% when applied to cotton fabric [293]. Significant antibacterial activity has been shown by cellulose nanocrystal (CN)–ZnO bio-nanocomposites against a variety of Gram-positive and Gram-negative bacteria, such as *Salmonella*, *Staphylococcus aureus*, and *Escherichia coli*. The virulence toxin genes are efficiently downregulated by these composites, which lessens the bacteria’s capacity to cause diseases [294]. In addition to increasing the antibacterial effectiveness, the combination of CNs with ZnO NPs also improves ZnO’s stability and dispersibility, both of which are essential for real-world applications [295]. For the first time, adding CNs/ZnO composites to chicken feed or using them as a coating material may be a useful way to lower the incidence of bacterial diseases and enhance the general health and productivity of poultry [296].

The CNC/ZnO nanocomposites can physically interact with bacterial cell walls, resulting in destruction of the structure. This interaction is facilitated by the nanocomposites’ high surface area and sharp edges, which disrupt the cell membranes, leading to the leakage of intracellular contents [294,297]. When ZnO NPs are exposed to light, they are known to produce reactive oxygen species (ROS), which can harm the lipids, proteins, and DNA of bacterial cells. One important component of CNC/ZnO nanocomposites’ antibacterial action is oxidative stress [297,298]. According to molecular docking studies, CNC/ZnO nanocomposites have the ability to block important bacterial enzymes that are essential for bacterial survival and replication, including dihydrofolate reductase (DHFR) and dihydropteroate synthetase (DHPS). This enzymatic inhibition further contributes to the antibacterial properties of the nanocomposites [298,299]. It has been demonstrated that the nanocomposites inhibit the expression of virulence genes in bacteria, hence decreasing the pathogenic potential of these organisms. These nanocomposites also have antibacterial properties through the control of gene expression [294].

By interfering with the extracellular polymeric materials that hold biofilms together, CNC/ZnO bio-nanocomposites have demonstrated effectiveness in inhibiting the formation of biofilms [294,300]. By penetrating biofilms and exhibiting antibacterial properties within the biofilm matrix, the bio-nanocomposites can decrease the viability of bacteria entrenched in biofilms [301]. Recent direct evidence has extended CNC/ZnO evaluation to PDR *C. perfringens* isolates recovered from poultry. Amin et al. [302] assessed both planktonic antimicrobial susceptibility and activity against established biofilms, thereby distinguishing antibacterial activity against free-living cells from effects on the biofilm phenotype. This represents a more relevant level of antibiofilm validation than studies demonstrating activity only against non-*C. perfringens* species; however, the evidence remains in vitro and requires confirmation in controlled poultry infection models before therapeutic or feed applications can be inferred.

Despite promising antimicrobial and antibiofilm activity, nanoparticle-based interventions require substantially more safety and translational evaluation before routine poultry application. Their biological behavior depends on composition, particle size, surface chemistry, dose, aggregation state, and route of administration. Potential concerns include cytotoxicity toward intestinal epithelial cells, accumulation or persistence of metallic components in edible tissues, disruption of beneficial gut microbial communities, and release of persistent nanomaterials into litter, soil, and water. Environmental exposure is particularly relevant for metal-based nanomaterials because repeated agricultural use may increase ecological metal loading and potentially contribute to co-selection pressures within environmental microbial communities. In addition, reproducible large-scale synthesis, batch-to-batch characterization, feed stability, cost-effectiveness, withdrawal requirements, residue limits, and regulatory approval remain unresolved for many experimental nano formulations. Accordingly, demonstration of in vitro antimicrobial or antibiofilm activity should be followed by pharmacokinetic, toxicological, residue-depletion, microbiome, environmental-fate, and controlled in vivo efficacy studies before field implementation.

The following Table 5 summarizes representative non-antibiotic interventions against *C. perfringens* in poultry, detailing their representative agents, experimental models, tested doses, primary outcomes, proposed mechanisms, and current limitations.

## 10. Future Perspectives and Research Gaps

Despite the considerable progress in understanding the pathogenesis and control of *C. perfringens* in poultry, several critical knowledge gaps remain. One of the major limitations is the incomplete understanding of the complex interplay between virulence factors, particularly toxin production [303], biofilm formation [151], and antimicrobial resistance [304]. While the *netB* gene has been widely recognized as a key virulence determinant, its presence alone does not fully explain disease occurrence, indicating that additional factors and regulatory mechanisms are involved.

Furthermore, the role of biofilm formation in *C. perfringens* pathogenicity is not yet fully elucidated. Although biofilms are known to enhance environmental persistence and resistance to antimicrobial agents, their direct contribution to disease severity and transmission under in vivo conditions requires further investigations. Standardized and reproducible methods for biofilm quantifications are also lacking, making it difficult to compare results across studies [156].

Another important gap lies in the variability of reported efficacy of alternative control strategies. Probiotics [21], prebiotics [305], organic acids [306], NPs, and AMPs [307] have shown promising results. However, their effectiveness is often strain-dependent and influenced by host factors, environmental conditions, and experimental design. There is a clear need for well-designed in vivo studies and large-scale trials to validate their approaches under field conditions [308].

In addition, limited information is available regarding the long-term safety, potential toxicity, and regulatory aspects of emerging interventions such as NPs and AMPs. The impact of these alternatives on the gut microbiome and their potential to induce resistance should also be carefully evaluated.

Future research should focus on integrative approaches combining genomics, transcriptomics, proteomics, and metabolomics to better understand the molecular mechanisms underlying *C. perfringens* virulence and adaptation [309]. Moreover, the development of target and synergistic strategies that simultaneously address toxin production, biofilm formation, and antimicrobial resistance may offer more effective and sustainable control of NE in poultry.

Overall, bridging these knowledge gaps will be essential for the development of innovative, safe, and effective alternatives to antibiotics in poultry production.

In conclusion, *C. perfringens*-associated NE remains a multifactorial challenge in poultry production in which toxin-mediated virulence, host susceptibility, intestinal dysbiosis, antimicrobial resistance, and biofilm-associated persistence can interact. Among non-antibiotic interventions, probiotics, particularly the selected *Bacillus* and *Lactobacillaceae* strains, together with several organic-acid, synbiotic, and phytogenic formulations, currently have the most substantial support from controlled poultry challenge studies. Bacteriophages and selected antimicrobial peptides have also demonstrated efficacy in experimental models, although delivery, spectrum, stability, and reproducibility remain important translational constraints. In contrast, nanoparticle and green-nanocomposite approaches show promising planktonic and antibiofilm activity but remain predominantly supported by in vitro evidence, with insufficient information on in vivo efficacy, residues, long-term safety, microbiome effects, environmental fate, and regulatory acceptability. Evidence for eradication of mature *C. perfringens* biofilms and large-scale commercial field efficacy remains limited across most intervention classes. Future studies should therefore prioritize standardized challenge and biofilm models, direct comparisons between planktonic and established-biofilm endpoints, well-controlled animal trials, and field validation incorporating safety, production performance, cost-effectiveness, and antimicrobial-resistance outcomes.

## Figures and Tables

**Table 1:** Reported economic and production impacts of necrotic enteritis across poultry-producing regions.

Region	Study Setting	Economic Parameters	Reported Effect	Evidence Type	Limitation	Reference
Global	Commercial poultry industry	Estimated annual economic burden	Approximately USD 6 billion annually due to production losses and disease-control costs	Global industry estimate/literature synthesis	These estimates are not based on a standardized multinational cost-of-illness analysis, as regional production costs and disease prevalence vary.	[16,17]
United States	Economic model of SNE using hypothetical 20,000-bird broiler flocks	Body weight, FCR, producer loss and additional feed cost	SNE modeling reduced body weight by 12% and increased FCR by 10.9%, driving estimated flock losses of USD 878.19–1480.52 alongside USD 370.49–739.38 in extra feed costs.	Economic modeling study	Rely on historical costs and an assumed 20% SNE incidence, meaning they cannot be directly applied to modern production systems.	[18]
Canada	Controlled broiler NE model	Mortality and intestinal lesions	Variant IBDV pre-exposure combined with *C. perfringens* challenge triggered 10–15% mortality and caused histopathological NE lesions in up to 70% of birds.	Experimental in vivo study	Controlled challenge model rather than commercial field-economic assessment	[14]
Australia	Controlled subclinical NE challenge in broilers	BWG and FCR	At day 16, NE challenge reduced BWG by 27% and worsened FCR by 11 points; by day 35, BWG remained 12% lower and FCR 5 points poorer than unchallenged controls	Experimental in vivo study	Focuses on performance loss rather than financial cost, while experimental challenge severity may not match all field outbreaks.	[19]
Egypt	Experimental *C. perfringens* infection in Cobb and Arbor Acres broilers	Growth, mortality, total return and economic efficiency	Untreated challenged birds had lower growth and returns than treated ones; Cobb birds yielded LE 39.02 when infected versus LE 44.91 when antibiotic-treated.	Experimental economic assessment	Reflects local, study-specific trial costs rather than nationwide economic burden.	[20]
China	Experimental *C. perfringens* challenge in broilers	Growth performance and intestinal injury	*C. perfringens* challenge significantly impaired growth performance together with intestinal barrier and inflammatory outcomes; dietary nisin mitigated these effects	Experimental in vivo study	Evaluates intervention efficacy rather than regional or national financial burden.	[15]

NE, necrotic enteritis; BWG, body-weight gain; FCR, food conversion rate; SNE, subclinical necrotic enteritis; IBDV, Infectious Bursal Disease Virus.

**Table 2:** Toxins and enzymes produced by *C. perfringens* in poultry.

Toxin Type	Synonym	Gene	Gene Location	Significant Effect	References
Alpha-toxin	Phospholipase C	*cpa*, *plc*	C	To hydrolyze cell membrane phospholipids	[3,27,28,29,30]
Perfringolysin O	θ-toxin (pore-forming toxin)	*pfoA*	C	Pore formation via binding to cholesterol-comprising cell membrane	[3,32,33,34]
Collagenase	κ-toxin	*colA*	C	To degrade collagen, which is the main component of the connective tissues of host cells	[3,31,35]
Sialidase	Secreted major neuraminidases	*nanI*	C	Involved in the removal of sialic acids from a variety of glycoconjugates on cell membranes	[24,36]
	Non-secreted neuraminidases	*nanH*	C		
	Secreted neuraminidases	*nanJ*	C		
Hyaluronidase	µ-toxin	*nagH*	C	To degrade hyaluronan coating cells, allowing direct contact between pathogen and host cells; or to degrade hyaluronan, leading to viscosity reduction, facilitating increased permeability of the connective tissues	[24,37]
		*nagI*	C		
		*nagJ*	C		
		*nagK*	C		
		*nagL*	C		
Collagen adhesion protein	Cell surface protein	*cnaA*	C	Binding of *C. perfringens* to collagen types IV, V and gelatine	[17,24]
NE B-like toxin	Pore-forming toxin	*netB*	P	To form heptameric, hydrophilic pores with a central diameter of approximately 26 Å	[22,38]
Beta2 toxin	Pore-forming toxin	*cpb2*	P	Pore-forming, leading to cell disruption	[22,39]
Toxin *C. perfringens* large cytotoxin	Large clostridial toxin	*tpeL*	P	Ras-specific glucosyl transferase activity, inactivating the Ras signaling pathway, leading to apoptosis	[22,40]

C, chromosome; P, plasmid; NE, necrotic enteritis.

**Table 3:** *C. perfringens* virulence factors.

Virulence Factor	Gene	Location	Functional Role	Evidence in Poultry NE	Interpretation	References
NetB	*netB*	Plasmid/NELoc-1	Pore formation	Strong experimental support	Major virulence determinant	[59,60,66]
CPA	*cpa/plc*	Chromosome	Phospholipase/membrane injury	Present, but not essential in knockout model	Contributory	[45,46,47]
PFO	*pfoA*	Chromosome	Cholesterol-dependent pore formation	Virulence-associated	Contributory	[48,49,50,70,71,72]
CPB2	*cpb2*	Plasmid	Cytotoxic/pore-forming activity	Association inconsistent	Candidate	[73,74,75,76,77,78,79,80]
TpeL	*tpeL*	Plasmid	Glucosylating toxin	Associated with increased severity in some strains	Virulence enhancer/candidate	[82,86,87,88,89,90]
CnaA	*cnaA*	Chromosome	Collagen adhesion	Experimental adhesion/virulence evidence	Adhesin	[92,93,94,95]
FimA/FimB	Corresponding pilus locus	Chromosome	Type IV pilus-mediated adhesion	Experimental evidence	Colonization	[94,95]
FbpA/FbpB	Respective loci	Chromosome	ECM/fibronectin interaction	Association/functional evidence	Adhesion	[16,96]
NanI/NanJ/NanH	*nanI*/*J*/*H*	Chromosome	Mucin/sialic-acid utilization	Mechanistic evidence	Nutrient acquisition/colonization	[97,98,99,100,101,102]
ZmpA/ZmpB	*zmpA*/*zmpB*	Plasmid/chromosome	Mucin degradation	Virulence attenuation evidence	Colonization/tissue invasion	[102,103,104,105]

NE, necrotic enteritis; ECM, extracellular matrix.

**Table 4:** Probiotics and necrotic enteritis prevention in poultry.

Genera	Bacteria (Probiotics)	Effect	Reference
*Lactobacillus*	*Lactobacillus acidophilus*	Controls the broiler microbiome and boosts beneficial microbes during *C. perfringens* exposure.	[178,179]
	*L. acidophilus* and *L. fermentum*	Reduce NE-induced inflammation, *C. perfringens* proliferation, and alpha-toxin production.	[180]
	*L. acidophilus* and *L. casei*	Decrease *C. perfringens* colonization, intestinal lesions, and NE-related mortality.	[181]
	*L. fermentum 1.2029*	Reduces NE lesion severity, IFN-γ levels, and toll-like receptor (TLR2) expression, while upregulating ileal IL-10.	[182]
	*L. johnsonii*	Suppresses *C. perfringens*-induced NE and exerts anti-inflammatory effects that protect broiler intestinal and liver tissues.	[183,184,185]
	*L. plantarum* and *L. rhamnosus*	Reduce pathogen-induced mortality along with intestinal *Salmonella* spp. and *C. perfringens* loads.	[186,187]
*Clostridium*	*Clostridium butyricum*	Stops NE progression in chickens by modulating immunity, barrier function, and gut microbiota, while improving weight gain and FCR.	[188,189]
*Enterococcus*	*Enterococcus faecium*	Optimizes gut microbiota composition, reduces inflammation, and enhances mucosal integrity in both healthy and *C. perfringens*-challenged broilers.	[190]
*Bacillus*	*Bacillus subtilis*	Improves bird performance, modulates gut microbiota and GALT activity, and prevents harmful bacterial colonization like *Clostridium* species.	[191,192,193,194]
	*Bacillus licheniformis*	Improves FCR, reinforces gut barrier integrity and GALT function, promotes balanced microbiota, produces bacteriocin-like compounds and lysozymes, and mitigates NE-associated oxidative stress.	[195,196,197]
	*Bacillus amyloliquefaciens*	Lowers pathogen load and lesion scores in *E. tenella-*, *E. maxima-* and *C. perfringens*-challenged broilers, while boosting *Lactobacillus* levels and FCR.	[198,199]
	*Weizmannia coagulans* (previously known as *Bacillus coagulans)*	Produces coagulin (a bacteriocin-like inhibitor) with potent activity against *C. perfringens*, inhibiting both its growth and alpha-toxin production.	[200,201,202,203]

α-toxin, alpha-toxin; IFNγ, interferon gamma; NE, necrotic enteritis; GALT, gut-associated lymphoid tissue; FCR, feed conversion ratio; *E. tenella*, *Eimeria tenella*.

**Table 5:** Comparative evidence for non-antibiotic interventions against *C. perfringens* and necrotic enteritis in poultry.

Intervention	Representative Agent	Experimental Model	Dose	Main Outcome	Proposed Mechanism	Evidence Level	Main Limitation	References
Probiotic	*Bacillus subtilis* PB6	*CP*-challenged broilers	10^7^ CFU/kg feed	Improved growth and intestinal barrier; reduced challenge-associated intestinal injury	Competitive exclusion, microbiota modulation, barrier support	In vivo	Strain- and challenge-model dependent	[172,173,174]
Prebiotic	Yeast β-glucan	NE-challenged broilers	200 mg/kg diet	Improved intestinal health, immune response and *CP* control	Immunomodulation, barrier support, microbiota modulation	In vivo	Product composition and host response vary	[217,218,219,220]
Organic acids	OA/EO formulation	NE-challenged broilers	Formulation-specific; report exact inclusion used in primary study	Improved intestinal integrity and reduced NE-associated effects	Intracellular acidification, membrane effects, microbiota modulation	In vivo	Efficacy depends strongly on formulation, coating and dose	[232]
Phytogenic	Thyme EO	*CP*-challenged broilers	0.25% diet	Reduced intestinal *CP* counts and intestinal lesions	Membrane disruption and antimicrobial activity	In vitro + in vivo	Composition varies among plant sources/batches	[275,276,277,278,279]
AMP	Sublancin	*CP* in vitro and challenged broilers	MIC: 8 μM in vitro; 2.88, 5.76, or 11.52 mg /L of drinking water in vivo, administered fresh daily for 7 days	Direct inhibition and amelioration of experimental NE	Membrane/cell-envelope damage	In vitro + in vivo	Stability, production cost, delivery and resistance require evaluation	[266]
Bacteriophage	*CP*-specific phage cocktail	Experimental broilers	Study-specific phage titre	Reduced experimental NE severity/mortality	Host-specific bacterial lysis	In vivo	Narrow host range, phage resistance and delivery stability	[245,246,247,248,249]
Ag NPs	Silver nanoparticles	MDR/XDR *CP* isolates from poultry and other animal/human sources	25–100 μg/mL for antibiofilm; 100 μg/mL for biofilm inhibition. MIC: 20 μg/mL; MBC: 30 μg/mL	Inhibition of biofilm formation	Membrane damage/oxidative stress	In vitro	Toxicity/residue/environmental uncertainty	[155,252]
CNC/ZnO	Cellulose nanocrystal/ZnO nanocomposite	PDR, biofilm-producing *CP* isolates recovered from chickens and turkeys	MIC: 0.125 μg/mL; MBC: 0.25 μg/mL	Planktonic inhibition and reduction of pre-existing biofilms	Cell-envelope injury; *agrB*/*pilA2* downregulation	In vitro	No equivalent therapeutic poultry/field validation	[302]

OA, organic acid; EO, essential oil; CP, *C. perfringens*; NE, necrotic enteritis; MDR, multidrug-resistant; XDR, extensive drug-resistant; PDR, pandrug-resistant; MIC, minimum inhibitory concentration; MBC, minimum bactericidal concentration.

## Data Availability

No new data were created or analyzed in this study. Data sharing is not applicable to this article.

## References

[1] Fathima S., Hakeem W., Shanmugasundaram R., Selvaraj R. (2022). Necrotic Enteritis in Broiler Chickens: A Review on the Pathogen, Pathogenesis, and Prevention. Microorganisms.

[2] Mamsin A., Barzani K.K.M., Mohammed B.T. (2024). Toxinotyping and Protein-Protein Interaction (PPI) Networks and Functional Annotation of the Detected Toxin Genes of *Clostridium perfringens* Isolated from food samples in Duhok Province, Iraq. Egypt. J. Vet. Sci..

[3] Rood J.I., Adams V., Lacey J., Lyras D., McClane B.A., Melville S.B., Moore R.J., Popoff M.R., Sarker M.R., Songer J.G. (2018). Expansion of the *Clostridium perfringens* toxin-based typing scheme. Anaerobe.

[4] Sattar M.M.K., Anjum A.A., Chang Y.F., Yaqub T., Aslam A., Ali T. (2023). Molecular Characterization and Toxins Optimization of Indigenous *Clostridium perfringens* Toxinotype B Isolated from Lamb Dysentery Clinical Cases. Kafkas Univ. Vet. Fak. Derg..

[5] Kiu R., Hall L.J. (2018). An update on the human and animal enteric pathogen *Clostridium perfringens*. Emerg. Microbes Infect..

[6] Cooper K.K., Songer J.G., Uzal F.A. (2013). Diagnosing clostridial enteric disease in poultry. J. Vet. Diagn. Investig..

[7] Alimolaei M., Ezatkhah M., Soltani S. (2023). Toxin genotypes of *Clostridium perfringens* isolates from common quail (*Coturnix coturnix*) with or without acute necrotic enteritis. Toxicon.

[8] Muneeb M., Khan E.U., Ahmad S., Naveed S., Ali M., Qazi M.A., Ahmad T., Abdollahi M.R. (2024). An updated review on alternative strategies to antibiotics against necrotic enteritis in commercial broiler chickens. World’s Poult. Sci. J..

[9] M’Sadeq S.A., Wu S., Swick R.A., Choct M. (2015). Towards the control of necrotic enteritis in broiler chickens with in-feed antibiotics phasing-out worldwide. Anim. Nutr..

[10] Shehata A.A., Basiouni S., Sting R., Akimkin V., Hoferer M., Hafez H.M. (2021). Poult Enteritis and Mortality Syndrome in Turkey Poults: Causes, Diagnosis and Preventive Measures. Animals.

[11] Riva S., Monjo T.P. (2021). The Major Predisposing Factors for Necrotic Enteritis in Broiler Chickens and the Use of Probiotics as New Strategy to Prevent the Disease. J. Vet. Med. Anim. Sci..

[12] Smits C.H., Li D., Patience J.F., den Hartog L. (2021). Animal Nutrition Strategies and Options to Reduce the Use of Antimicrobials in Animal Production.

[13] Khodambashi Emami N. (2020). Managing Poultry Gut Integrity, Immunity and Microbial Balance During Necrotic Enteritis. Ph.D. Thesis.

[14] Gautam H., Ayalew L.E., Shaik N.A., Subhasinghe I., Popowich S., Chow-Lockerbie B., Dixon A., Ahmed K.A., Tikoo S.K., Gomis S. (2024). Exploring the predictive power of jejunal microbiome composition in clinical and subclinical necrotic enteritis caused by *Clostridium perfringens*: Insights from a broiler chicken model. J. Transl. Med..

[15] Yuan H., Bai G., Lin Y., Yu X., Yang Q., Dou R., Sun H., Zhao Z., Li Z., Chen Z. (2024). Effects of dietary Nisin on growth performance, immune function and gut health of broilers challenged by *Clostridium perfringens*. J. Anim. Sci..

[16] Emami N.K., Dalloul R.A. (2021). Centennial Review: Recent developments in host-pathogen interactions during necrotic enteritis in poultry. Poult. Sci..

[17] Wade B., Keyburn A.L., Seemann T., Rood J.I., Moore R.J. (2015). Binding of *Clostridium perfringens* to collagen correlates with the ability to cause necrotic enteritis in chickens. Vet. Microbiol..

[18] Skinner J.T., Bauer S., Young V., Pauling G., Wilson J. (2010). An economic analysis of the impact of subclinical (mild) necrotic enteritis in broiler chickens. Avian Dis..

[19] Keerqin C., Rhayat L., Zhang Z.-H., Gharib-Naseri K., Kheravii S., Devillard E., Crowley T., Wu S.-B. (2021). Probiotic *Bacillus subtilis* 29,784 improved weight gain and enhanced gut health status of broilers under necrotic enteritis condition. Poult. Sci..

[20] Sallam E.A., Mohammed L.S., Elbasuni S.S., Azam A.E., Soliman M.M. (2021). Impacts of Microbial based Therapy on Growth Performance, Intestinal Health, Carcass Traits and Economic Efficiency of *Clostridium perfringens*-Infected Cobb and Arbor Acres Broilers. Vet. Med. Sci..

[21] Kulkarni R.R., Gaghan C., Gorrell K., Sharif S., Taha-Abdelaziz K. (2022). Probiotics as alternatives to antibiotics for the prevention and control of necrotic enteritis in chickens. Pathogens.

[22] El-Hack M.E.A., El-Saadony M.T., Elbestawy A.R., El-Shall N.A., Saad A.M., Salem H.M., El-Tahan A.M., Khafaga A.F., Taha A.E., AbuQamar S.F. (2022). Necrotic enteritis in broiler chickens: Disease characteristics and prevention using organic antibiotic alternatives—A comprehensive review. Poult. Sci..

[23] Moore R.J. (2024). Necrotic enteritis and antibiotic-free production of broiler chickens: Challenges in testing and using alternative products. Anim. Nutr..

[24] Mehdizadeh Gohari I., Navarro M.A., Li J., Shrestha A., Uzal F., McClane B.A. (2021). Pathogenicity and virulence of *Clostridium perfringens*. Virulence.

[25] Wade B., Keyburn A.L., Haring V., Ford M., Rood J.I., Moore R.J. (2016). The adherent abilities of *Clostridium perfringens* strains are critical for the pathogenesis of avian necrotic enteritis. Vet. Microbiol..

[26] Mohiuddin M., Song Z., Liao S., Qi N., Li J., Lv M., Lin X., Cai H., Hu J., Liu S. (2023). Animal Model Studies, Antibiotic Resistance and Toxin Gene Profile of NE Reproducing *Clostridium perfringens* Type A and Type G Strains Isolated from Commercial Poultry Farms in China. Microorganisms.

[27] Akram A., Hassani I.T., Khan M.A., Taj M.K. (2023). Pathogenicity of *Clostridium perfringens*: A Short Review. Pak.-Euro J. Med. Life Sci..

[28] Grenda T., Jarosz A., Sapała M., Grenda A., Patyra E., Kwiatek K. (2023). *Clostridium perfringens*—Opportunistic Foodborne Pathogen, Its Diversity and Epidemiological Significance. Pathogens.

[29] Ferreira M.R.A., Moreira G.M.S., Cunha C.E.P.D., Mendonça M., Salvarani F.M., Moreira Â.N., Conceição F.R. (2016). Recombinant alpha, beta, and epsilon toxins of *Clostridium perfringens*: Production strategies and applications as veterinary vaccines. Toxins.

[30] Monturiol-Gross L., Villalta-Romero F., Flores-Díaz M., Alape-Girón A. (2021). Bacterial phospholipases C with dual activity: Phosphatidylcholinesterase and sphingomyelinase. FEBS Open Bio.

[31] Lee K.-W., Lillehoj H.S. (2021). Role of *Clostridium perfringens* necrotic enteritis B-like toxin in disease pathogenesis. Vaccines.

[32] Popoff M.R. (2014). Clostridial pore-forming toxins: Powerful virulence factors. Anaerobe.

[33] Wang G., Liu Y., Deng L., Liu H., Deng X., Li Q., Feng H., Guo Z., Qiu J. (2023). Repurposing rabeprazole sodium as an anti-*Clostridium perfringens* drug by inhibiting perfringolysin O. J. Appl. Microbiol..

[34] Kuo J., Uzunovic J., Jacobson A., Dourado M., Gierke S., Rajendram M., Keilberg D., Mar J., Stekol E., Curry J. (2024). Toxigenic *Clostridium perfringens* isolated from at-risk paediatric inflammatory bowel disease patients. J. Crohn’s Colitis.

[35] Garvey M. (2023). Foodborne Clostridioides Species: Pathogenicity, Virulence and Biocontrol Options. Microorganisms.

[36] Wang Y.-H. (2020). Sialidases from *Clostridium perfringens* and their inhibitors. Front. Cell. Infect. Microbiol..

[37] Goossens E., Valgaeren B.R., Pardon B., Haesebrouck F., Ducatelle R., Deprez P.R., Van Immerseel F. (2017). Rethinking the role of alpha toxin in *Clostridium perfringens*-associated enteric diseases: A review on bovine necro-haemorrhagic enteritis. Vet. Res..

[38] Savva C.G., da Costa S.P.F., Bokori-Brown M., Naylor C.E., Cole A.R., Moss D.S., Titball R.W., Basak A.K. (2013). Molecular architecture and functional analysis of NetB, a pore-forming toxin from *Clostridium perfringens*. J. Biol. Chem..

[39] Uzal F.A., Freedman J.C., Shrestha A., Theoret J.R., Garcia J., Awad M.M., Adams V., Moore R.J., Rood J.I., McClane B.A. (2014). Towards an understanding of the role of *Clostridium perfringens* toxins in human and animal disease. Future Microbiol..

[40] Chen J., McClane B.A. (2015). Characterization of *Clostridium perfringens* TpeL toxin gene carriage, production, cytotoxic contributions, and trypsin sensitivity. Infect. Immun..

[41] Revitt-Mills S.A., Rood J.I., Adams V. (2015). *Clostridium perfringens* extracellular toxins and enzymes: 20 and counting. Microbiol. Aust..

[42] Oda M., Terao Y., Sakurai J., Nagahama M. (2015). Membrane-binding mechanism of *Clostridium perfringens* alpha-toxin. Toxins.

[43] Navarro M.A., McClane B.A., Uzal F.A. (2018). Mechanisms of action and cell death associated with *Clostridium perfringens* toxins. Toxins.

[44] Revitt-Mills S.A., Vidor C.J., Watts T.D., Lyras D., Rood J.I., Adams V. (2019). Virulence plasmids of the pathogenic Clostridia. Microbiol. Spectr..

[45] Helal S.S., Khalaf N.M., El Menisy A.A., Lebdah M.A. (2019). *Clostridium perfringens* type A causing necrotic enteritis outbreaks among chickens in Egypt. Zagazig Vet. J..

[46] Fu Y., Alenezi T., Sun X. (2022). *Clostridium perfringens*-induced necrotic diseases: An overview. Immuno.

[47] Uzal F.A., Giannitti F., Asin J. (2022). Yellow Lamb Disease (*Clostridium perfringens* Type A Enterotoxemia of Sheep): A Review. Animals.

[48] Forti K., Cagiola M., Pellegrini M., Anzalone L., Di Paolo A., Corneli S., Severi G., De Giuseppe A. (2020). Generation of recombinant baculovirus expressing atoxic C-terminal CPA toxin of *Clostridium perfringens* and production of specific antibodies. BMC Biotechnol..

[49] Rasool S., Hussain T., Khan S.M., Zehra A., Tahreem S., Kakroo A.M. (2017). Toxins of *Clostridium perfringens* as virulence factors in animal diseases. J. Pharmacogn. Phytochem..

[50] Suzaki A., Ohtani K., Komine-Aizawa S., Matsumoto A., Kamiya S., Hayakawa S. (2021). Pathogenic characterization of *Clostridium perfringens* strains isolated from patients with massive intravascular hemolysis. Front. Microbiol..

[51] Keyburn A.L., Sheedy S.A., Ford M.E., Williamson M.M., Awad M.M., Rood J.I., Moore R.J. (2006). Alpha-toxin of *Clostridium perfringens* is not an essential virulence factor in necrotic enteritis in chickens. Infect. Immun..

[52] Li Y., Li Y., Mengist H.M., Shi C., Zhang C., Wang B., Li T., Huang Y., Xu Y., Jin T. (2021). Structural basis of the pore-forming toxin/membrane interaction. Toxins.

[53] Yan X.-X., Porter C.J., Hardy S.P., Steer D., Smith A.I., Quinsey N.S., Hughes V., Cheung J.K., Keyburn A.L., Kaldhusdal M. (2013). Structural and functional analysis of the pore-forming toxin NetB from *Clostridium perfringens*. mBio.

[54] Prescott J.F., Parreira V.R., Mehdizadeh Gohari I., Lepp D., Gong J. (2016). The pathogenesis of necrotic enteritis in chickens: What we know and what we need to know: A review. Avian Pathol..

[55] Rood J.I., Keyburn A.L., Moore R.J. (2016). NetB and necrotic enteritis: The hole movable story. Avian Pathol..

[56] Ohtani K., Shimizu T. (2016). Regulation of toxin production in *Clostridium perfringens*. Toxins.

[57] Mora Z.V.-D.L., Macías-Rodríguez M.E., Arratia-Quijada J., Gonzalez-Torres Y.S., Nuño K., Villarruel-López A. (2020). *Clostridium perfringens* as foodborne pathogen in broiler production: Pathophysiology and potential strategies for controlling necrotic enteritis. Animals.

[58] Yu Q., Lepp D., Mehdizadeh Gohari I., Wu T., Zhou H., Yin X., Yu H., Prescott J.F., Nie S.-P., Xie M.-Y. (2017). The Agr-like quorum sensing system is required for pathogenesis of necrotic enteritis caused by *Clostridium perfringens* in poultry. Infect. Immun..

[59] Keyburn A.L., Boyce J.D., Vaz P., Bannam T.L., Ford M.E., Parker D., Di Rubbo A., Rood J.I., Moore R.J. (2008). NetB, a new toxin that is associated with avian necrotic enteritis caused by *Clostridium perfringens*. PLoS Pathog..

[60] Keyburn A.L., Yan X.X., Bannam T.L., Van Immerseel F., Rood J.I., Moore R.J. (2010). Association between avian necrotic enteritis and *Clostridium perfringens* strains expressing NetB toxin. Vet. Res..

[61] Sarmah H., Hazarika R., Tamuly S., Deka P., Manoharan S., Sharma R.K. (2021). Evaluation of different antigenic preparations against necrotic enteritis in broiler birds using a novel *Clostridium perfringens* type G strain. Anaerobe.

[62] Cooper K.K., Songer J.G. (2010). Virulence of *Clostridium perfringens* in an experimental model of poultry necrotic enteritis. Vet. Microbiol..

[63] Shini S., Zhang D., Aland R., Li X., Dart P., Callaghan M., Speight R., Bryden W. (2020). Probiotic *Bacillus amyloliquefaciens* H57 ameliorates subclinical necrotic enteritis in broiler chicks by maintaining intestinal mucosal integrity and improving feed efficiency. Poult. Sci..

[64] Goossens E., Dierick E., Ducatelle R., Van Immerseel F. (2020). Spotlight on Avian Pathology: Untangling Contradictory Disease Descriptions of Necrotic Enteritis and Necro-Haemorrhagic Enteritis in Broilers.

[65] Abildgaard L., Sondergaard T.E., Engberg R.M., Schramm A., Højberg O. (2010). In vitro production of necrotic enteritis toxin B, NetB, by netB-positive and netB-negative *Clostridium perfringens* originating from healthy and diseased broiler chickens. Vet. Microbiol..

[66] Deslauriers N., Maduro L., Lepp D., Gong J., Abdul-Careem M.F., Boulianne M. (2024). Determination of the virulence status of *Clostridium perfringens* strains using a chicken intestinal ligated loop model is important for understanding the pathogenesis of necrotic. Poult. Sci..

[67] Lacey J.A., Johanesen P.A., Lyras D., Moore R.J. (2016). Genomic diversity of necrotic enteritis-associated strains of *Clostridium perfringens*: A review. Avian Pathol..

[68] Li C., Lillehoj H.S., Gadde U.D., Ritter D., Oh S. (2017). Characterization of *Clostridium perfringens* strains isolated from healthy and necrotic enteritis-afflicted broiler chickens. Avian Dis..

[69] Verherstraeten S., Goossens E., Valgaeren B., Pardon B., Timbermont L., Haesebrouck F., Ducatelle R., Deprez P., Wade K.R., Tweten R. (2015). Perfringolysin O: The underrated *Clostridium perfringens* toxin?. Toxins.

[70] Morton C.J., Sani M.-A., Parker M.W., Separovic F. (2019). Cholesterol-dependent cytolysins: Membrane and protein structural requirements for pore formation: Focus review. Chem. Rev..

[71] Mc Guinness C. (2022). Perfringolysin O Pore Formation Dynamics: From Soluble Monomer to Membrane Insertion and Beyond. Ph.D. Thesis.

[72] Shewell L.K., Day C.J., Jen F.E.-C., Haselhorst T., Atack J.M., Reijneveld J.F., Everest-Dass A., James D.B., Boguslawski K.M., Brouwer S. (2020). All major cholesterol-dependent cytolysins use glycans as cellular receptors. Sci. Adv..

[73] Simpson K.M., Callan R.J., Van Metre D.C. (2018). Clostridial abomasitis and enteritis in ruminants. Vet. Clin. Food Anim. Pract..

[74] Gao X., Yang Q., Huang X., Yan Z., Zhang S., Luo R., Wang P., Wang W., Xie K., Jiang T. (2020). Effects of *Clostridium perfringens* beta2 toxin on apoptosis, inflammation, and barrier function of intestinal porcine epithelial cells. Microb. Pathog..

[75] Dierick E., Goossens E., Prescott J.F., Ducatelle R., Van Immerseel F. (2022). Enteric Clostridia. Pathogenesis of Bacterial Infections in Animals.

[76] Mehdizadeh Gohari I. (2018). Understanding Type A *Clostridium perfringens* in Foal Necrotizing Enteritis and Canine Acute Hemorrhagic Diarrhea. Ph.D. Thesis.

[77] Forti K., Ferroni L., Pellegrini M., Cruciani D., De Giuseppe A., Crotti S., Papa P., Maresca C., Severi G., Marenzoni M.L. (2020). Molecular characterization of *Clostridium perfringens* strains isolated in Italy. Toxins.

[78] Freedman J.C., Theoret J.R., Wisniewski J.A., Uzal F.A., Rood J.I., McClane B.A. (2015). *Clostridium perfringens* type A–E toxin plasmids. Res. Microbiol..

[79] Park MiSeon P.M., Rafii F. (2019). The prevalence of plasmid-coded cpe enterotoxin, β2 toxin, tpeL toxin, and tetracycline resistance in *Clostridium perfringens* strains isolated from different sources. Anaerobe.

[80] Benz R., Piselli C., Hoxha C., Koy C., Glocker M.O., Popoff M.R. (2022). *Clostridium perfringens* Beta2 toxin forms highly cation-selective channels in lipid bilayers. Eur. Biophys. J..

[81] Saadat A.P. (2021). Analysis of TpeL Secretion in *Clostridium perfringens*. Ph.D. Thesis.

[82] Svobodová I., Hulánková R. (2024). Nontyping virulence factors of *Clostridium perfringens*. Acta Vet. Brno.

[83] Schöttelndreier D., Langejürgen A., Lindner R., Genth H. (2020). Low density lipoprotein receptor-related protein-1 (LRP1) is involved in the uptake of Clostridioides difficile toxin A and serves as an internalizing receptor. Front. Cell. Infect. Microbiol..

[84] Orrell K.E. (2021). Biochemical and Genomic Insights into the Translocation of Large Clostridial Toxins Across Host Cell Membranes. Ph.D. Thesis.

[85] Zhou R., He L., Zhang J., Zhang X., Li Y., Zhan X., Tao L. (2024). Molecular basis of TMPRSS2 recognition by *Paeniclostridium sordellii* hemorrhagic toxin. Nat. Commun..

[86] Saadat A., Melville S.B. (2021). Holin-dependent secretion of the large clostridial toxin TpeL by *Clostridium perfringens*. J. Bacteriol..

[87] Khiav L.A., Zahmatkesh A., Paradise A. (2023). Detection of clostridium perfringens types through genetic profiling and mouse neutralisation test. Bulg. J. Vet. Med..

[88] Gu C., Lillehoj H.S., Sun Z., Lee Y., Zhao H., Xianyu Z., Yan X., Wang Y., Lin S., Liu L. (2019). Characterization of virulent netB+/tpeL+ *Clostridium perfringens* strains from necrotic enteritis–affected broiler chicken farms. Avian Dis..

[89] Orrell K.E., Melnyk R.A. (2021). Large clostridial toxins: Mechanisms and roles in disease. Microbiol. Mol. Biol. Rev..

[90] Fancher C.A., Thames H.T., Colvin M.G., Zhang L., Nuthalapati N., Kiess A., Dinh T.T., Sukumaran A.T. (2021). Research Note: Prevalence and molecular characteristics of *Clostridium perfringens* in “no antibiotics ever” broiler farms. Poult. Sci..

[91] Kiu R., Brown J., Bedwell H., Leclaire C., Caim S., Pickard D., Dougan G., Dixon R.A., Hall L.J. (2019). Genomic analysis on broiler-associated *Clostridium perfringens* strains and exploratory caecal microbiome investigation reveals key factors linked to poultry necrotic enteritis. Anim. Microbiome.

[92] Goo D., Park I., Nam H., Lee Y., Sawall J., Smith A., Rehberger T., Li C., Lillehoj H. (2023). Collagen adhesin protein and necrotic enteritis B-like toxin as biomarkers for early diagnosis of necrotic enteritis in commercial broiler chickens. Poult. Sci..

[93] Zhou Y., Lepp D., Carere J., Yu H., Yang C., Gong J. (2021). A Novel PilR/PilS Two-Component System Regulates Necrotic Enteritis Pilus Production in *Clostridium perfringens*. J. Bacteriol..

[94] Zhou Y. (2022). Regulation of Pilus Production by the PilR/PilS Two-Component and Agr-Like Quorum Sensing Systems in *Clostridium perfringens*. Master’s Thesis.

[95] Lepp D., Zhou Y., Ojha S., Mehdizadeh Gohari I., Carere J., Yang C., Prescott J., Gong J. (2021). *Clostridium perfringens* produces an adhesive pilus required for the pathogenesis of necrotic enteritis in poultry. J. Bacteriol..

[96] Speziale P., Arciola C.R., Pietrocola G. (2019). Fibronectin and its role in human infective diseases. Cells.

[97] Lewis A.L., Chen X., Schnaar R.L., Varki A. (2022). Sialic acids and other nonulosonic acids. Essentials of Glycobiology.

[98] Juge N., Tailford L., Owen C.D. (2016). Sialidases from gut bacteria: A mini-review. Biochem. Soc. Trans..

[99] Sudhakara P., Sellamuthu I., Aruni A.W. (2019). Bacterial sialoglycosidases in virulence and pathogenesis. Pathogens.

[100] Yimer L. (2022). Major virulence factors and pathogenicity islands in pathogenic clostridium species. Asian J. Microbiol. Biotechnol..

[101] Medley B. (2023). Investigating the Molecular Basis of Substrate Specificity in the Sialidase NanH and O-glycopeptidase Amuc_1438 Involved in Mucin Degradation. Ph.D. Thesis.

[102] Martens E.C., Neumann M., Desai M.S. (2018). Interactions of commensal and pathogenic microorganisms with the intestinal mucosal barrier. Nat. Rev. Microbiol..

[103] Wade B., Keyburn A.L., Haring V., Ford M., Rood J.I., Moore R.J. (2020). Two putative zinc metalloproteases contribute to the virulence of *Clostridium perfringens* strains that cause avian necrotic enteritis. J. Vet. Diagn. Investig..

[104] Van Leuven N. (2021). Towards a Better Understanding of Factors Influencing NetB Cytotoxicity: An In Vitro Study. Master’s Thesis.

[105] Caminero A., Guzman M., Libertucci J., Lomax A.E. (2023). The emerging roles of bacterial proteases in intestinal diseases. Gut Microbes.

[106] Ke C.H., Wu C.E., Lin F., Yang W.Y. (2025). Differential gene expression in *Clostridium perfringens* during pre-and post-infection phases and in jejunal tissues of broilers with necrotic enteritis induced by *Clostridium perfringens* alone and its coinfection with Eimeria. Poult. Sci..

[107] Qin S., Xiao W., Zhou C., Pu Q., Deng X., Lan L., Liang H., Song X., Wu M. (2022). Pseudomonas aeruginosa: Pathogenesis, virulence factors, antibiotic resistance, interaction with host, technology advances and emerging therapeutics. Signal Transduct. Target. Ther..

[108] Zaragoza N.E., Orellana C.A., Moonen G.A., Moutafis G., Marcellin E. (2019). Vaccine production to protect animals against pathogenic clostridia. Toxins.

[109] Gieryńska M., Szulc-Dąbrowska L., Struzik J., Mielcarska M.B., Gregorczyk-Zboroch K.P. (2022). Integrity of the intestinal barrier: The involvement of epithelial cells and microbiota—A mutual relationship. Animals.

[110] Low K.E., Smith S.P., Abbott D.W., Boraston A.B. (2021). The glycoconjugate-degrading enzymes of *Clostridium perfringens*: Tailored catalysts for breaching the intestinal mucus barrier. Glycobiology.

[111] Qu D., Wang G., Yu L., Tian F., Chen W., Zhai Q. (2021). The effects of diet and gut microbiota on the regulation of intestinal mucin glycosylation. Carbohydr. Polym..

[112] Lacey J.A., Keyburn A.L., Ford M.E., Portela R.W., Johanesen P.A., Lyras D., Moore R.J. (2017). Conjugation-mediated horizontal gene transfer of *Clostridium perfringens* plasmids in the chicken gastrointestinal tract results in the formation of new virulent strains. Appl. Environ. Microbiol..

[113] He W. (2021). A Natural Model of Immunomodulation for Necrotic Enteritis in Poultry. Master’s Thesis.

[114] MacMillan J.L., Vicaretti S.D., Noyovitz B., Xing X., Low K.E., Inglis G.D., Zaytsoff S.J., Boraston A.B., Smith S.P., Uwiera R.R. (2019). Structural analysis of broiler chicken small intestinal mucin O-glycan modification by *Clostridium perfringens*. Poult. Sci..

[115] Paiva D., McElroy A. (2014). Necrotic enteritis: Applications for the poultry industry. J. Appl. Poult. Res..

[116] Parreira V.R., Russell K., Athanasiadou S., Prescott J.F. (2016). Comparative transcriptome analysis by RNAseq of necrotic enteritis *Clostridium perfringens* during in vivo colonization and in vitro conditions. BMC Microbiol..

[117] Olkowski A., Wojnarowicz C., Chirino-Trejo M., Drew M. (2006). Responses of broiler chickens orally challenged with *Clostridium perfringens* isolated from field cases of necrotic enteritis. Res. Vet. Sci..

[118] Vidal J.E., Shak J.R., Canizalez-Roman A. (2015). The CpAL quorum sensing system regulates production of hemolysins CPA and PFO to build *Clostridium perfringens* biofilms. Infect. Immun..

[119] Liu Z., Mou S., Li L., Chen Q., Yang R., Guo S., Jin Y., Liu L., Li T., Chen H. (2024). The barrier disruption and pyroptosis of intestinal epithelial cells caused by perfringolysin O (PFO) from *Clostridium perfringens*. Cells.

[120] Gharib-Naseri K., Kheravii S., Keerqin C., Swick R.A., Choct M., Wu S.B. (2021). Differential expression of intestinal genes in necrotic enteritis challenged broiler chickens with 2 different *Clostridium perfringens* strains. Poult. Sci..

[121] Baril S.A., Wilson K.A., Shaik M.M., Fukuda Y., Umans R.A., Barbieri A., Lynch J., Gose T., Myasnikov A., Oldham M.L. (2024). The role of ATP-binding Cassette subfamily B member 6 in the inner ear. Nat. Commun..

[122] Wang C., Peng Y., Yang H., Jiang Y., Khalid A.K., Zhang K., Xie S., Bermudez L., Yang Y., Zhang L. (2025). RBMX2 links *Mycobacterium bovis* infection to epithelial–mesenchymal transition and lung cancer progression. eLife.

[123] Wang C., Jiang Y., Yang Z., Xu H., Khalid A.K., Iftakhar T., Peng Y., Lu L., Zhang L., Bermudez L. (2024). Host factor RBMX2 promotes epithelial cell apoptosis by downregulating APAF-1’s Retention Intron after *Mycobacterium bovis* infection. Front. Immunol..

[124] Calik A., Niraula A., Dong B., Blue C.E.C., Fenster D.A., Dalloul R.A. (2024). Iohexol-based assessment of intestinal permeability in broilers challenged with Eimeria maxima, *Clostridium perfringens* or both. Front. Physiol..

[125] Goo D., Ko H., Sharma M.K., Choppa V.S.R., Paneru D., Shi H., Kim W.K. (2024). Comparison of necrotic enteritis effects on growth performance and intestinal health in two different meat-type chicken strains Athens Canadian Random Bred and Cobb 500. Poult. Sci..

[126] Aqeel M., Mirani A.H., Khoso P.A., Sahito J.K., Bhutto A.L., Leghari R.A., Rahimoon M.M., Ali K., Ali N. (2024). A review on the study of immunomodulators and herbal remedies: A natural approach to treating necrotic enteritis. Pure Appl. Biol. (PAB).

[127] Yudiarti T., Sugiharto S., Widiastuti E., Wahyuni H., Sartono T., Nasution M. (2024). Physiological parameters, intestinal microbial population and internal organ weight of broilers supplemented with the fungus monascus purpureus. Adv. Anim. Vet. Sci..

[128] Soromou L.W., Leno P.F., Kamano A., Souare M.L., Camara A.O.D., Camara K. (2024). Current practices in the veterinary use of antibiotics in poultry laying hens in Friguiagbé (Guinea). J. Drug Deliv. Ther..

[129] Taha S.M., Abd El-Aziz N.K., Abdelkhalek A., Pet I., Ahmadi M., El-Nabtity S.M. (2024). Chitosan-*Loaded Lagenaria siceraria* and *Thymus vulgaris* potentiate antibacterial, antioxidant, and immunomodulatory activities against extensive drug-resistant *Pseudomonas aeruginosa* and vancomycin-resistant *Staphylococcus aureus*: In vitro and in vivo approaches. Antioxidants.

[130] Góchez D., Raicek M., Pinto Ferreira J., Jeannin M., Moulin G., Erlacher-Vindel E. (2019). OIE annual report on antimicrobial agents intended for use in animals: Methods used. Front. Vet. Sci..

[131] García-Vela S., Martínez-Sancho A., Said L.B., Torres C., Fliss I. (2023). Pathogenicity and Antibiotic Resistance Diversity in *Clostridium perfringens* Isolates from Poultry Affected by Necrotic Enteritis in Canada. Pathogens.

[132] Gomaa N.H., El-Aziz N.K.A., El-Naenaeey E.Y., Abdelaziz W.S., Sewid A.H. (2023). Antimicrobial potential of myricetin-coated zinc oxide nanocomposite against drug-resistant *Clostridium perfringens*. BMC Microbiol..

[133] Bendary M.M., Abd El-Hamid M.I., El-Tarabili R.M., Hefny A.A., Algendy R.M., Elzohairy N.A., Ghoneim M.M., Al-Sanea M.M., Nahari M.H., Moustafa W.H. (2022). *Clostridium perfringens* Associated with Foodborne Infections of Animal Origins: Insights into Prevalence, Antimicrobial Resistance, Toxin Genes Profiles, and Toxinotypes. Biology.

[134] Eman M., Sharaf D.M. (2024). Comparing the effect of nitazoxanide and tylosin against necrotic enteritis in broilers. J. Adv. Vet. Res..

[135] Lu R., Liu B., Wu L., Bao H., García P., Wang Y., Zhou Y., Zhang H. (2023). A broad-spectrum phage endolysin (LysCP28) able to remove biofilms and inactivate *Clostridium perfringens* strains. Foods.

[136] Ray S., Löffler S., Richter-Dahlfors A. (2024). High-Resolution Large-Area Image Analysis Deciphers the Distribution of Salmonella Cells and ECM Components in Biofilms Formed on Charged PEDOT: PSS Surfaces. Adv. Sci..

[137] Pantaléon V., Bouttier S., Soavelomandroso A.P., Janoir C., Candela T. (2014). Biofilms of Clostridium species. Anaerobe.

[138] Kelly S.M., Lanigan N., O’Neill I.J., Bottacini F., Lugli G.A., Viappiani A., Turroni F., Ventura M., van Sinderen D. (2020). Bifidobacterial biofilm formation is a multifactorial adaptive phenomenon in response to bile exposure. Sci. Rep..

[139] Freitas A.I., Lopes N., Oliveira F., Brás S., França Â., Vasconcelos C., Vilanova M., Cerca N. (2018). Comparative analysis between biofilm formation and gene expression in *Staphylococcus epidermidis* isolates. Future Microbiol..

[140] Charlebois A., Jacques M., Archambault M. (2014). Biofilm formation of *Clostridium perfringens* and its exposure to low-dose antimicrobials. Front. Microbiol..

[141] Varga J.J., Therit B., Melville S.B. (2008). Type IV pili and the CcpA protein are needed for maximal biofilm formation by the gram-positive anaerobic pathogen *Clostridium perfringens*. Infect. Immun..

[142] De Oliveira A., Cataneli Pereira V., Pinheiro L., Moraes Riboli D.F., Benini Martins K., Ribeiro de Souza da Cunha M.D.L. (2016). Antimicrobial resistance profile of planktonic and biofilm cells of *Staphylococcus aureus* and coagulase-negative staphylococci. Int. J. Mol. Sci..

[143] Li P., Yin R., Cheng J., Lin J. (2023). Bacterial biofilm formation on biomaterials and approaches to its treatment and prevention. Int. J. Mol. Sci..

[144] Yu S., Su T., Wu H., Liu S., Wang D., Zhao T., Jin Z., Du W., Zhu M.-J., Chua S.L. (2015). PslG, a self-produced glycosyl hydrolase, triggers biofilm disassembly by disrupting exopolysaccharide matrix. Cell Res..

[145] Czuban M., Srinivasan S., Yee N.A., Agustin E., Koliszak A., Miller E., Khan I., Quinones I., Noory H., Motola C. (2018). Bio-orthogonal chemistry and reloadable biomaterial enable local activation of antibiotic prodrugs and enhance treatments against *Staphylococcus aureus* infections. ACS Cent. Sci..

[146] Tian S., Su L., Liu Y., Cao J., Yang G., Ren Y., Huang F., Liu J., An Y., van der Mei H.C. (2020). Self-targeting, zwitterionic micellar dispersants enhance antibiotic killing of infectious biofilms—An intravital imaging study in mice. Sci. Adv..

[147] Bellich B., Lagatolla C., Tossi A., Benincasa M., Cescutti P., Rizzo R. (2018). Influence of bacterial biofilm polysaccharide structure on interactions with antimicrobial peptides: A study on *Klebsiella pneumoniae*. Int. J. Mol. Sci..

[148] Geng X., Yang Y.-J., Li Z., Ge W.-B., Xu X., Liu X.-W., Li J.-Y. (2024). Fingolimod Inhibits Exopolysaccharide Production and Regulates Relevant Genes to Eliminate the Biofilm of *K. pneumoniae*. Int. J. Mol. Sci..

[149] Bai X., Nakatsu C.H., Bhunia A.K. (2021). Bacterial biofilms and their implications in pathogenesis and food safety. Foods.

[150] Jamal M., Ahmad W., Andleeb S., Jalil F., Imran M., Nawaz M.A., Hussain T., Ali M., Rafiq M., Kamil M.A. (2018). Bacterial biofilm and associated infections. J. Chin. Med. Assoc..

[151] Hu W.S., Woo D.U., Kang Y.J., Koo O.K. (2021). Biofilm and spore formation of *Clostridium perfringens* and its resistance to disinfectant and oxidative stress. Antibiotics.

[152] Charlebois A., Jacques M., Archambault M. (2016). Comparative transcriptomic analysis of *Clostridium perfringens* biofilms and planktonic cells. Avian Pathol..

[153] Zhang X., Ma Y., Ye G. (2018). Morphological observation and comparative transcriptomic analysis of *Clostridium perfringens* biofilm and planktonic cells. Curr. Microbiol..

[154] Gharieb R., Saad M., Abdallah K., Khedr M., Farag E., Abd El-Fattah A. (2021). Insights on toxin genotyping, virulence, antibiogram profiling, biofilm formation and efficacy of disinfectants on biofilms of *Clostridium perfringens* isolated from poultry, animals and humans. J. Appl. Microbiol..

[155] Ahmed H.A., El Bayomi R.M., Hamed R.I., Mohsen R.A., El-Gohary F.A., Hefny A.A., Elkhawaga E., Tolba H.M. (2022). Genetic relatedness, antibiotic resistance, and effect of silver nanoparticle on biofilm formation by *Clostridium perfringens* isolated from chickens, pigeons, camels, and human consumers. Vet. Sci..

[156] Bhattrai S., Sun Z., Jenkins M., Parker C., Lillehoj H., Li C. (2026). Biofilm formation by clinical *Clostridium perfringens* isolates and its suppression by thymol. Poult. Sci..

[157] Toyofuku M., Inaba T., Kiyokawa T., Obana N., Yawata Y., Nomura N. (2016). Environmental factors that shape biofilm formation. Biosci. Biotechnol. Biochem..

[158] Condinho M., Carvalho B., Cruz A., Pinto S.N., Arraiano C.M., Pobre V. (2023). The role of RNA regulators, quorum sensing and c-di-GMP in bacterial biofilm formation. FEBS Open Bio.

[159] Elumalai P., Gao X., Cui J., Kumar A.S., Dhandapani P., Parthipan P., Karthikeyan O.P., Theerthagiri J., Kheawhom S., Choi M.Y. (2024). Biofilm formation, occurrence, microbial communication, impact and characterization methods in natural and anthropic systems: A review. Environ. Chem. Lett..

[160] Alotaibi G.F., Bukhari M.A. (2021). Factors influencing bacterial biofilm formation and development. Am. J. Biomed. Sci. Res..

[161] Rather M.A., Gupta K., Mandal M. (2021). Microbial biofilm: Formation, architecture, antibiotic resistance, and control strategies. Braz. J. Microbiol..

[162] Dsouza F.P., Dinesh S., Sharma S. (2024). Understanding the intricacies of microbial biofilm formation and its endurance in chronic infections: A key to advancing biofilm-targeted therapeutic strategies. Arch. Microbiol..

[163] Paul S.S., Rama Rao S.V., Hegde N., Williams N.J., Chatterjee R.N., Raju M.V.L.N., Reddy G.N., Kumar V., Phani Kumar P.S., Mallick S. (2022). Effects of dietary antimicrobial growth promoters on performance parameters and abundance and diversity of broiler chicken gut microbiome and selection of antibiotic resistance genes. Front. Microbiol..

[164] Cella E., Giovanetti M., Benedetti F., Scarpa F., Johnston C., Borsetti A., Ceccarelli G., Azarian T., Zella D., Ciccozzi M. (2023). Joining forces against antibiotic resistance: The one health solution. Pathogens.

[165] Islam R., Sultana N., Bhakta S., Haque Z., Hasan A., Siddique M.P., Islam M.R. (2023). Modulation of growth performance, gut morphometry, and cecal microbiota in broilers by clove (*Syzygium aromaticum*) and tulsi (*Ocimum sanctum*) supplementation. Poult. Sci..

[166] El-Ghany A., Wafaa A. (2024). The influence of the phytobiotic tulsi (*Ocimum sanctum*) on broiler production and health. Vet. Stanica.

[167] Ciurescu G., Dumitru M., Gheorghe A., Untea A., Drăghici R. (2020). Effect of *Bacillus subtilis* on growth performance, bone mineralization, and bacterial population of broilers fed with different protein sources. Poult. Sci..

[168] Khalique A., Zeng D., Shoaib M., Wang H., Qing X., Rajput D.S., Pan K., Ni X. (2020). Probiotics mitigating subclinical necrotic enteritis (SNE) as potential alternatives to antibiotics in poultry. AMB Express.

[169] Jadhav K., Sharma K., Katoch S., Sharma V., Mane B. (2015). Probiotics in broiler poultry feeds: A review. J. Anim. Nutr. Physiol..

[170] Golnari M., Bahrami N., Milanian Z., Rabbani Khorasgani M., Asadollahi M.A., Shafiei R., Fatemi S.S.-A. (2024). Isolation and characterization of novel Bacillus strains with superior probiotic potential: Comparative analysis and safety evaluation. Sci. Rep..

[171] Monteiro C.R., do Carmo M.S., Melo B.O., Alves M.S., Dos Santos C.I., Monteiro S.G., Bomfim M.R.Q., Fernandes E.S., Monteiro-Neto V. (2019). In vitro antimicrobial activity and probiotic potential of *Bifidobacterium* and *Lactobacillus* against species of Clostridium. Nutrients.

[172] El-Sayed Y., Khalil W., Fayez N., Mohamed Abdel-Fattah A.-F. (2024). Enhancing effect of oregano essential oil and *Bacillus subtilis* on broiler immune function, intestinal morphology and growth performance. BMC Vet. Res..

[173] Mushtaq M., Ali B., Ali M., BiBi N., Raut R., Suliman G.M., Swelum A.A. (2024). Different levels of single-strain probiotic (*Bacillus subtilis*) with proteolytic enzyme (serratiopeptidase) can be used as an alternative to antibiotic growth promoters in broiler. Poult. Sci..

[174] Thirumeignanam D., Chellapandian M., Arulnathan N., Parthiban S., Kumar V., Vijayakumar M.P., Chauhan S. (2024). Evaluation of Natural Antimicrobial Substances Blend as a Replacement for Antibiotic Growth Promoters in Broiler Chickens: Enhancing Growth and Managing Intestinal Bacterial Diseases. Curr. Microbiol..

[175] Liu Y., Zhang S., Luo Z., Liu D. (2021). Supplemental *Bacillus subtilis* PB6 improves growth performance and gut health in broilers challenged with *Clostridium perfringens*. J. Immunol. Res..

[176] Chen P., Lv H., Du M., Liu W., Che C., Zhao J., Liu H. (2024). *Bacillus subtilis* HW2 enhances growth performance and alleviates gut injury via attenuation of endoplasmic reticulum stress and regulation of gut microbiota in broilers under necrotic enteritis challenge. Poult. Sci..

[177] Fancher C.A., Zhang L., Kiess A.S., Adhikari P.A., Dinh T.T., Sukumaran A.T. (2020). Avian pathogenic Escherichia coli and *Clostridium perfringens*: Challenges in no antibiotics ever broiler production and potential solutions. Microorganisms.

[178] Li Z., Wang W., Liu D., Guo Y. (2017). Effects of Lactobacillus acidophilus on gut microbiota composition in broilers challenged with *Clostridium perfringens*. PLoS ONE.

[179] Pertiwi H., Mahendra M.Y.N. (2021). Probiotic *Lactobacillus* sp. Improved Performance of Broiler Chicken: A Review. Syst. Rev. Pharm..

[180] Guo S., Liu D., Zhang B., Li Z., Li Y., Ding B., Guo Y. (2017). Two Lactobacillus species inhibit the growth and α-toxin production of *Clostridium perfringens* and induced proinflammatory factors in chicken intestinal epithelial cells in vitro. Front. Microbiol..

[181] Emami N.K., Calik A., White M.B., Young M., Dalloul R.A. (2019). Necrotic enteritis in broiler chickens: The role of tight junctions and mucosal immune responses in alleviating the effect of the disease. Microorganisms.

[182] Vieco-Saiz N., Belguesmia Y., Raspoet R., Auclair E., Padgett C., Bailey C., Gancel F., Drider D. (2022). Protective effects of novel *Lactobacillaceae* strains isolated from chicken Caeca against necrotic enteritis infection: In vitro and in vivo evidences. Microorganisms.

[183] Wang H., Ni X., Qing X., Liu L., Lai J., Khalique A., Li G., Pan K., Jing B., Zeng D. (2017). Probiotic enhanced intestinal immunity in broilers against subclinical necrotic enteritis. Front. Immunol..

[184] Wang H., Ni X., Qing X., Liu L., Xin J., Luo M., Khalique A., Dan Y., Pan K., Jing B. (2018). Probiotic *Lactobacillus johnsonii* BS15 improves blood parameters related to immunity in broilers experimentally infected with subclinical necrotic enteritis. Front. Microbiol..

[185] Khalique A., Zeng D., Wang H., Qing X., Zhou Y., Xin J., Zeng Y., Pan K., Shu G., Jing B. (2019). Transcriptome analysis revealed ameliorative effect of probiotic *Lactobacillus johnsonii* BS15 against subclinical necrotic enteritis induced hepatic inflammation in broilers. Microb. Pathog..

[186] Kupryś-Caruk M., Michalczuk M., Chabłowska B., Stefańska I., Kotyrba D., Parzeniecka-Jaworska M. (2018). Efficacy and safety assessment of microbiological feed additive for chicken broilers in tolerance studies. J. Vet. Res..

[187] Michalczuk M., Holl E., Möddel A., Jóźwik A., Słósarz B., Nie J., Żąbek D., Konieczka K. (2021). Phytogenic Ingredients from Hops and Organic Acids Improve Selected Indices of Welfare, Health Status Markers, and Bacteria Composition in the Caeca of Broiler Chickens. Animals.

[188] Huang T., Peng X.-Y., Gao B., Wei Q.-L., Xiang R., Yuan M.-G., Xu Z.-H. (2019). The effect of *Clostridium butyricum* on gut microbiota, immune response and intestinal barrier function during the development of necrotic enteritis in chickens. Front. Microbiol..

[189] Liu M., Uyanga V.A., Cao X., Liu X., Lin H. (2023). Regulatory effects of the probiotic *Clostridium butyricum* on gut microbes, intestinal health, and growth performance of chickens. J. Poult. Sci..

[190] Wu Y., Zhen W., Geng Y., Wang Z., Guo Y. (2019). Pretreatment with probiotic *Enterococcus faecium* NCIMB 11181 ameliorates necrotic enteritis-induced intestinal barrier injury in broiler chickens. Sci. Rep..

[191] Al-Baadani H.H., Abudabos A.M., Al-Mufarrej S.I., Al-Baadani A.A., Alhidary I.A. (2018). Dietary supplementation of *Bacillus subtilis*, *Saccharomyces cerevisiae* and their symbiotic effect on serum biochemical parameters in broilers challenged with *Clostridium perfringens*. J. Appl. Anim. Res..

[192] Sokale A., Menconi A., Mathis G., Lumpkins B., Sims M., Whelan R., Doranalli K. (2019). Effect of *Bacillus subtilis* DSM 32315 on the intestinal structural integrity and growth performance of broiler chickens under necrotic enteritis challenge. Poult. Sci..

[193] Zhao Y., Zeng D., Wang H., Qing X., Sun N., Xin J., Luo M., Khalique A., Pan K., Shu G. (2020). Dietary probiotic *Bacillus licheniformis* H2 enhanced growth performance, morphology of small intestine and liver, and antioxidant capacity of broiler chickens against *Clostridium perfringens*-induced subclinical necrotic enteritis. Probiotics Antimicrob. Proteins.

[194] Nerber H.N., Sorg J.A. (2024). The small acid-soluble proteins of spore-forming organisms: Similarities and differences in function. Anaerobe.

[195] Zhou M., Zeng D., Ni X., Tu T., Yin Z., Pan K., Jing B. (2016). Effects of *Bacillus licheniformis* on the growth performance and expression of lipid metabolism-related genes in broiler chickens challenged with *Clostridium perfringens*-induced necrotic enteritis. Lipids Health Dis..

[196] Xu S., Lin Y., Zeng D., Zhou M., Zeng Y., Wang H., Zhou Y., Zhu H., Pan K., Jing B. (2018). *Bacillus licheniformis* normalize the ileum microbiota of chickens infected with necrotic enteritis. Sci. Rep..

[197] Ogbuewu I.P., Mabelebele M., Sebola N.A., Mbajiorgu C. (2022). Bacillus probiotics as alternatives to in-feed antibiotics and its influence on growth, serum chemistry, antioxidant status, intestinal histomorphology, and lesion scores in disease-challenged broiler chickens. Front. Vet. Sci..

[198] Tsukahara T., Inoue R., Nakayama K., Inatomi T. (2018). Inclusion of *Bacillus amyloliquefaciens* strain TOA 5001 in the diet of broilers suppresses the symptoms of coccidiosis by modulating intestinal microbiota. Anim. Sci. J..

[199] Luise D., Bosi P., Raff L., Amatucci L., Virdis S., Trevisi P. (2022). *Bacillus* spp. probiotic strains as a potential tool for limiting the use of antibiotics, and improving the growth and health of pigs and chickens. Front. Microbiol..

[200] Kawarizadeh A., Tabatabaei M., Hosseinzadeh S., Farzaneh M., Pourmontaseri M. (2019). The effects of probiotic *Bacillus coagulans* on the cytotoxicity and expression of alpha toxin gene of *Clostridium perfringens* type A. Anaerobe.

[201] Fijan S., Fijan T., Connil N. (2023). Overview of probiotic strains of Weizmannia coagulans, previously known as *Bacillus coagulans*, as food supplements and their use in human health. Appl. Microbiol..

[202] Yeni F., Samut H., Soyer Y. (2023). Effect of Non-LAB Probiotics on Foodborne Enteric Pathogens: A Systematic Review. Food Rev. Int..

[203] Zhang S., Li P., Lee S., Wang Y., Tan C., Shang N. (2024). Weizmannia coagulans: An ideal probiotic for gut health. Food Sci. Hum. Wellness.

[204] You S., Ma Y., Yan B., Pei W., Wu Q., Ding C., Huang C. (2022). The promotion mechanism of prebiotics for probiotics: A review. Front. Nutr..

[205] Biswas S., Kim M.H., Baek D.H., Kim I.H. (2023). Probiotic mixture (*Bacillus subtilis* and *Bacillus licheniformis*) a potential in-feed additive to improve broiler production efficiency, nutrient digestibility, caecal microflora, meat quality and to diminish hazardous odour emission. J. Anim. Physiol. Anim. Nutr..

[206] Zhang Z., Wen Z., Huang X., Haifang X., Xiaomin S., Chen L., Zhang B., Jiale Y., Yuhong L., Changwu Y. (2023). Research Progress of Rare Carbohydrate Prebiotics. Sci. Technol. Food Ind..

[207] Peng M., Tabashsum Z., Anderson M., Truong A., Houser A.K., Padilla J., Akmel A., Bhatti J., Rahaman S.O., Biswas D. (2020). Effectiveness of probiotics, prebiotics, and prebiotic-like components in common functional foods. Compr. Rev. Food Sci. Food Saf..

[208] Dong S., Li L., Hao F., Fang Z., Zhong R., Wu J., Fang X. (2024). Improving quality of poultry and its meat products with probiotics, prebiotics, and phytoextracts. Poult. Sci..

[209] Barbalho R.L.D.C., Castaneda C., Araújo L.F., Kiess A.S., Carvalho R.S., Barbalho C.B., Borges L.L., Bonato M.A. (2023). Β-glucans and MOS, essential oil, and probiotics in diets of broilers challenged with *Eimeria* spp. and *Clostridium perfringens*. Poult. Sci..

[210] Ahiwe E., Dos Santos T.T., Graham H., Iji P. (2021). Can probiotic or prebiotic yeast (*Saccharomyces cerevisiae*) serve as alternatives to in-feed antibiotics for healthy or disease-challenged broiler chickens?: A review. J. Appl. Poult. Res..

[211] Aziz A.A.A., Aziz E.-S.A.A., Khairy M.H., Abdelaziz A.S. (2024). Insights on the use of butyric acid, and nucleotides as feed additives in poultry: A review. J. Adv. Vet. Res..

[212] Pascual A., Pauletto M., Giantin M., Radaelli G., Ballarin C., Birolo M., Zomeño C., Dacasto M., Bortoletti M., Vascellari M. (2020). Effect of dietary supplementation with yeast cell wall extracts on performance and gut response in broiler chickens. J. Anim. Sci. Biotechnol..

[213] Rawling M., Schiavone M., Apper E., Merrifield D.L., Castex M., Leclercq E., Foey A. (2023). Yeast cell wall extracts from *Saccharomyces cerevisiae* varying in structure and composition differentially shape the innate immunity and mucosal tissue responses of the intestine of zebrafish (*Danio rerio*). Front. Immunol..

[214] Zhang T., Cheng T., Geng S., Mao K., Li X., Gao J., Han J., Sang Y. (2024). Synbiotic Combination between Lactobacillus paracasei VL8 and Mannan-Oligosaccharide Repairs the Intestinal Barrier in the Dextran Sulfate Sodium-Induced Colitis Model by Regulating the Intestinal Stem Cell Niche. J. Agric. Food Chem..

[215] Zhang X., Xu H., Gong L., Wang J., Fu J., Lv Z., Zhou L., Li X., Liu Q., Xia P. (2024). Mannanase improves the growth performance of broilers by alleviating inflammation of the intestinal epithelium and improving intestinal microbiota. Anim. Nutr..

[216] Poudel I., Adhikari P. (2024). Combating the persistence of Salmonella infections in laying hens: Nutritional, managemental and vaccination strategies. World’s Poult. Sci. J..

[217] Marchi P.H., Vendramini T.H.A., Zafalon R.V.A., Príncipe L.D.A., Cesar C.G.L., Perini M.P., Putarov T.C., Gomes C.O.M.S., Balieiro J.C.D.C., Brunetto M.A. (2024). Effects of Increasing Levels of Purified Beta-1, 3/1, 6-Glucans on the Fecal Microbiome, Digestibility, and Immunity Variables of Healthy Adult Dogs. Microorganisms.

[218] Tian X., Shao Y., Wang Z., Guo Y. (2016). Effects of dietary yeast β-glucans supplementation on growth performance, gut morphology, intestinal *Clostridium perfringens* population and immune response of broiler chickens challenged with necrotic enteritis. Anim. Feed Sci. Technol..

[219] He W., Kamely M., Wakaruk J., Goes E.C., Korver D.R., Barreda D.R. (2023). Early-life β-glucan exposure enhances disease resilience of broiler chickens to a natural *Clostridium perfringens* infection. Dev. Comp. Immunol..

[220] Kyoung H., Kim E., Cho J.H., Lee H., Kim Y., Park K.I., Kim H.B., Song M. (2023). Dietary yeast cell wall enhanced intestinal health of broiler chickens by modulating intestinal integrity, immune responses, and microbiota. Poult. Sci..

[221] Li C., Niu Z., Zou M., Liu S., Wang M., Gu X., Lu H., Tian H., Jha R. (2020). Probiotics, prebiotics, and synbiotics regulate the intestinal microbiota differentially and restore the relative abundance of specific gut microorganisms. J. Dairy Sci..

[222] Razali F.N., Teoh W.Y., Ramli M.Z., Loo C.-Y., Gnanaraj C. (2024). Role of prebiotics, probiotics, and synbiotics in the management of colonic disorders. Advanced Drug Delivery Systems for Colonic Disorders.

[223] Shanmugasundaram R., Markazi A., Mortada M., Ng T., Applegate T., Bielke L., Syed B., Pender C., Curry S., Murugesan G. (2020). Research Note: Effect of synbiotic supplementation on caecal *Clostridium perfringens* load in broiler chickens with different necrotic enteritis challenge models. Poult. Sci..

[224] Shah B.R., Hakeem W.A., Shanmugasundaram R., Selvaraj R.K. (2023). Effect of synbiotic supplementation on production performance and severity of necrotic enteritis in broilers during an experimental necrotic enteritis challenge. Poult. Sci..

[225] Krueger L., Spangler D., Vandermyde D., Sims M., Ayangbile G. (2017). Avi-Lution^®^ supplemented at 1.0 or 2.0 g/kg in feed improves the growth performance of broiler chickens during challenge with bacitracin-resistant *Clostridium perfringens*. Poult. Sci..

[226] Shahidi S., Yahyavi M., Zare D. (2014). Influence of dietary organic acids supplementation on reproductive performance of freshwater Angelfish (*Pterophyllum scalare*). Glob. Vet..

[227] Baltić B., Starčević M., Đorđević J., Mrdović B., Marković R. (2017). Importance of medium chain fatty acids in animal nutrition. IOP Conf. Ser. Earth Environ. Sci..

[228] Szabó R.T., Kovács-Weber M., Zimborán Á., Kovács L., Erdélyi M. (2023). Effects of Short-and Medium-Chain Fatty Acids on Production, Meat Quality, and Microbial Attributes—A Review. Molecules.

[229] Ben Braïek O., Smaoui S. (2021). Chemistry, safety, and challenges of the use of organic acids and their derivative salts in meat preservation. J. Food Qual..

[230] Dai D., Qiu K., Zhang H.-J., Wu S.-G., Han Y.-M., Wu Y.-Y., Qi G.-H., Wang J. (2021). Organic acids as alternatives for antibiotic growth promoters alter the intestinal structure and microbiota and improve the growth performance in broilers. Front. Microbiol..

[231] Abeer E., Barakat M., Ali G.I., Naena N.A., Salim A.A. (2020). Effect of some antibiotic alternatives on experimentally Escherichia coli infected broiler chicks. Alex. J. Veter-Sci..

[232] Kumar A., Toghyani M., Kheravii S.K., Pineda L., Han Y., Swick R.A., Wu S.-B. (2022). Organic acid blends improve intestinal integrity, modulate short-chain fatty acids profiles and alter microbiota of broilers under necrotic enteritis challenge. Anim. Nutr..

[233] Granstad S., Kristoffersen A.B., Benestad S.L., Sjurseth S.K., David B., Sørensen L., Fjermedal A., Edvardsen D.H., Sanson G., Løvland A. (2020). Effect of feed additives as alternatives to in-feed antimicrobials on production performance and intestinal *Clostridium perfringens* counts in broiler chickens. Animals.

[234] Abdelli N., Pérez J.F., Vilarrasa E., Cabeza Luna I., Melo-Duran D., D’Angelo M., Solà-Oriol D. (2020). Targeted-release organic acids and essential oils improve performance and digestive function in broilers under a necrotic enteritis challenge. Animals.

[235] Appleton S.R., Ballou A., Watkins K.L. (2024). Use of Monoglycerides and Diglycerides to Mitigate Poultry Production Losses: A Review. Vet. Sci..

[236] Gomez-Osorio L.-M., Yepes-Medina V., Ballou A., Parini M., Angel R. (2021). Short and medium chain fatty acids and their derivatives as a natural strategy in the control of necrotic enteritis and microbial homeostasis in broiler chickens. Front. Vet. Sci..

[237] Tugnoli B., Giovagnoni G., Piva A., Grilli E. (2020). From acidifiers to intestinal health enhancers: How organic acids can improve growth efficiency of pigs. Animals.

[238] Nhara R.B., Marume U., Nantapo C.W.T. (2024). Potential of Organic Acids, Essential Oils and Their Blends in Pig Diets as Alternatives to Antibiotic Growth Promoters. Animals.

[239] Dittoe D.K., Ricke S.C., Kiess A.S. (2018). Organic Acids and Potential for Modifying the Avian Gastrointestinal Tract and Reducing Pathogens and Disease. Front. Vet. Sci..

[240] Ranveer S.A., Dasriya V., Ahmad M.F., Dhillon H.S., Samtiya M., Shama E., Anand T., Dhewa T., Chaudhary V., Chaudhary P. (2024). Positive and negative aspects of bacteriophages and their immense role in the food chain. npj Sci. Food.

[241] Shkoporov A.N., Turkington C.J., Hill C. (2022). Mutualistic interplay between bacteriophages and bacteria in the human gut. Nat. Rev. Microbiol..

[242] Chevallereau A., Pons B.J., van Houte S., Westra E.R. (2022). Interactions between bacterial and phage communities in natural environments. Nat. Rev. Microbiol..

[243] Jiang A., Liu Z., Lv X., Zhou C., Ran T., Tan Z. (2024). Prospects and Challenges of Bacteriophage Substitution for Antibiotics in Livestock and Poultry Production. Biology.

[244] Moye Z.D., Woolston J., Sulakvelidze A. (2018). Bacteriophage applications for food production and processing. Viruses.

[245] Mohammadi T.N., Shen C., Li Y., Zayda M.G., Sato J., Masuda Y., Honjoh K.-I., Miyamoto T. (2022). Characterization and optimization of bacteriophage cocktails to control *Clostridium perfringens* in vitro and in curry roux. Int. J. Food Microbiol..

[246] Tian Y., Wu L., Lu R., Bao H., Zhou Y., Pang M., Brown J., Wang J., Wang R., Zhang H. (2022). Virulent phage vB_CpeP_HN02 inhibits *Clostridium perfringens* on the surface of the chicken meat. Int. J. Food Microbiol..

[247] Thanki A.M., Osei E.K., Whenham N., Salter M.G., Bedford M.R., Masey O’Neill H.V., Clokie M.R. (2024). Broad host range phages target global *Clostridium perfringens* bacterial strains and clear infection in five-strain model systems. Microbiol. Spectr..

[248] Mohd Shaufi M.A., Sieo C.C., Chong C.W., Tan G.H., Omar A.R., Ho Y.W. (2024). Multiple factorial analysis of growth performance, gut population, lipid profiles, immune responses, intestinal histomorphology, and relative organ weights of Cobb 500 broilers fed a diet supplemented with phage cocktail and probiotics. Ital. J. Anim. Sci..

[249] Hosny R.A., Gaber A.F., Sorour H.K. (2021). Bacteriophage mediated control of necrotic enteritis caused by *C. perfringens* in broiler chickens. Vet. Res. Commun..

[250] Swain P.S., Prusty S., Rao S.B.N., Rajendran D., Patra A.K. (2021). Essential nanominerals and other nanomaterials in poultry nutrition and production. Advances in Poultry Nutrition Research.

[251] Patra A. (2019). Are nanomaterials potential new generation antimicrobial feed additives in livestock. Indian J. Anim. Heal.

[252] Torky H.A., Khalil S.A., Elkassas F.A., Rezk M.S., Tawfik R.G. (2023). Effect of Silver Nanoparticles on Biofilm Formation by *Clostridium perfringens* Isolated from Poultry and Molecular Typing of Strains by ERIC-PCR. J. Adv. Vet. Res..

[253] Line J.E., Seal B.S., Garrish J.K. (2022). Selected antimicrobial peptides inhibit in vitro growth of *Campylobacter* spp. Appl. Microbiol..

[254] Rodrigues G., Souza Santos L., Franco O.L. (2022). Antimicrobial Peptides Controlling Resistant Bacteria in Animal Production. Front. Microbiol..

[255] Li Y., Slavik K.M., Toyoda H.C., Morehouse B.R., de Oliveira Mann C.C., Elek A., Levy S., Wang Z., Mears K.S., Liu J. (2023). CGLRs are a diverse family of pattern recognition receptors in innate immunity. Cell.

[256] Gallardo-Becerra L., Cervantes-Echeverría M., Cornejo-Granados F., Vazquez-Morado L.E., Ochoa-Leyva A. (2024). Perspectives in Searching Antimicrobial Peptides (AMPs) Produced by the Microbiota. Microb. Ecol..

[257] Roque-Borda C.A., Pereira L.P., Guastalli E.A.L., Soares N.M., Mac-Lean P.A.B., Salgado D.D.A., Meneguin A.B., Chorilli M., Vicente E.F. (2021). Hpmcp-coated microcapsules containing the ctx (Ile21)-ha antimicrobial peptide reduce the mortality rate caused by resistant Salmonella enteritidis in laying hens. Antibiotics.

[258] Mulukutla A., Shreshtha R., Deb V.K., Chatterjee P., Jain U., Chauhan N. (2024). Recent Advances in antimicrobial peptide-based therapy. Bioorg. Chem..

[259] Egido J.E., Costa A.R., Aparicio-Maldonado C., Haas P.-J., Brouns S.J. (2022). Mechanisms and clinical importance of bacteriophage resistance. FEMS Microbiol. Rev..

[260] Pontes J.T.C.D., Toledo Borges A.B., Roque-Borda C.A., Pavan F.R. (2022). Antimicrobial peptides as an alternative for the eradication of bacterial biofilms of multi-drug resistant bacteria. Pharmaceutics.

[261] Magana M., Pushpanathan M., Santos A.L., Leanse L., Fernandez M., Ioannidis A., Giulianotti M.A., Apidianakis Y., Bradfute S., Ferguson A.L. (2020). The value of antimicrobial peptides in the age of resistance. Lancet Infect. Dis..

[262] Abreu R., Semedo-Lemsaddek T., Cunha E., Tavares L., Oliveira M. (2023). Antimicrobial drug resistance in poultry production: Current status and innovative strategies for bacterial control. Microorganisms.

[263] Daneshmand A., Kermanshahi H., Sekhavati M.H., Javadmanesh A., Ahmadian M. (2019). Antimicrobial peptide, cLF36, affects performance and intestinal morphology, microflora, junctional proteins, and immune cells in broilers challenged with *E. coli*. Sci. Rep..

[264] García-Vela S., Guay L.-D., Rahman M.R.T., Biron E., Torres C., Fliss I. (2024). Antimicrobial Activity of Synthetic Enterocins A, B, P, SEK4, and L50, Alone and in Combinations, against *Clostridium perfringens*. Int. J. Mol. Sci..

[265] Hernández-González J.C., Martínez-Tapia A., Lazcano-Hernández G., García-Pérez B.E., Castrejón-Jiménez N.S. (2021). Bacteriocins from lactic acid bacteria. A powerful alternative as antimicrobials, probiotics, and immunomodulators in veterinary medicine. Animals.

[266] Wang S., Zeng X., Wang Q., Zhu J., Peng Q., Hou C., Thacker P., Qiao S. (2015). The antimicrobial peptide sublancin ameliorates necrotic enteritis induced by *Clostridium perfringens* in broilers. J. Anim. Sci..

[267] Kierończyk B., Rawski M., Mikołajczak Z., Świątkiewicz S., Józefiak D. (2020). Nisin as a novel feed additive: The effects on gut microbial modulation and activity, histological parameters, and growth performance of broiler chickens. Animals.

[268] Kierończyk B., Pruszyńska-Oszmałek E., Świątkiewicz S., Rawski M., Długosz J., Engberg R., Józefiak D. (2016). The nisin improves broiler chicken growth performance and interacts with salinomycin in terms of gastrointestinal tract microbiota composition. J. Anim. Feed Sci..

[269] Rafeeq M., Bilal R.M., Batool F., Yameen K., Farag M.R., Madkour M., Elnesr S.S., El-Shall N.A., Dhama K., Alagawany M. (2023). Application of herbs and their derivatives in broiler chickens: A review. World’s Poult. Sci. J..

[270] Ipçak H.H. (2023). The Role of Phytogenic Feed Additives in Modulating Poultry Nutritional Physiology and Genomics.

[271] Biswas S., Ahn J.M., Kim I.H. (2024). Assessing the potential of phytogenic feed additives: A comprehensive review on their effectiveness as a potent dietary enhancement for nonruminant in swine and poultry. J. Anim. Physiol. Anim. Nutr..

[272] Djuragic O., Čabarkapa I., Šeremešić M.M., Rakita S., Tomičić Z. (2023). Feed Additives, Their Role, and Technological Properties. Sustainable Use of Feed Additives in Livestock: Novel Ways for Animal Production.

[273] Li Y., Kong D., Fu Y., Sussman M.R., Wu H. (2020). The effect of developmental and environmental factors on secondary metabolites in medicinal plants. Plant Physiol. Biochem..

[274] Čabarkapa I., Puvača N., Popović S., Čolović D., Kostadinović L., Tatham E.K., Lević J. (2020). Aromatic plants and their extracts pharmacokinetics and in vitro/in vivo mechanisms of action. Feed Additives.

[275] Yang Y., Wang Q., Diarra M.S., Yu H., Hua Y., Gong J. (2016). Functional assessment of encapsulated citral for controlling necrotic enteritis in broiler chickens. Poult. Sci..

[276] Zhang L., Peng Q., Liu Y., Ma Q., Zhang J., Guo Y., Xue Z., Zhao L. (2021). Effects of oregano essential oil as an antibiotic growth promoter alternative on growth performance, antioxidant status, and intestinal health of broilers. Poult. Sci..

[277] Noruzi S., Torki M., Mohammadi H. (2022). Effects of supplementing diet with Thyme (*Thymuas vulgaris* L.) essential oil and/or selenium yeast on production performance and blood variables of broiler chickens. Vet. Med. Sci..

[278] Abd El-Hack M.E., El-Saadony M.T., Saad A.M., Salem H.M., Ashry N.M., Ghanima M.M.A., Shukry M., Swelum A.A., Taha A.E., El-Tahan A.M. (2022). Essential oils and their nanoemulsions as green alternatives to antibiotics in poultry nutrition: A comprehensive review. Poult. Sci..

[279] Gharaibeh M.H., Khalifeh M.S., Nawasreh A.N., Hananeh W.M., Awawdeh M.S. (2021). Assessment of immune response and efficacy of essential oils application on controlling necrotic enteritis induced by *Clostridium perfringens* in broiler chickens. Molecules.

[280] Li S., Zhang K., Bai S., Wang J., Zeng Q., Peng H., Lv H., Mu Y., Xuan Y., Li S. (2024). Extract of *Scutellaria baicalensis* and *Lonicerae flos* Improves Growth Performance, Antioxidant Capacity, and Intestinal Barrier of Yellow-Feather Broiler Chickens Against *Clostridium perfringens*. Poult. Sci..

[281] Gómez-García M., Argüello H., Puente H., Mencía-Ares Ó., González S., Miranda R., Rubio P., Carvajal A. (2020). In-depth in vitro evaluation of the activity and mechanisms of action of organic acids and essential oils against swine enteropathogenic bacteria. Front. Vet. Sci..

[282] Huang X., Lao Y., Pan Y., Chen Y., Zhao H., Gong L., Xie N., Mo C.-H. (2021). Synergistic antimicrobial effectiveness of plant essential oil and its application in seafood preservation: A review. Molecules.

[283] van Eerden E., Santos R.R., Molist F., Dardi M., Pantoja-Millas L.A., Molist-Badiola J., Baratelli M., Pages M. (2022). Efficacy of an attenuated vaccine against avian coccidiosis in combination with feed additives based on organic acids and essential oils on production performance and intestinal lesions in broilers experimentally challenged with necrotic enteritis. Poult. Sci..

[284] Pham V.H., Abbas W., Huang J., Guo F., Zhang K., Kong L., Zhen W., Guo Y., Wang Z. (2023). Dietary coated essential oil and organic acid mixture supplementation improves health of broilers infected with avian pathogenic Escherichia coli. Anim. Nutr..

[285] Qiao J., Shang Z., Liu X., Wang K., Wu Z., Wei Q., Li H. (2022). Regulatory effects of combined dietary supplementation with essential oils and organic acids on microbial communities of cobb broilers. Front. Microbiol..

[286] Huang J., Guo F., Abbas W., Hu Z., Liu L., Qiao J., Bi R., Xu T., Zhang K., Huang J. (2024). Effects of microencapsulated essential oils and organic acids preparation on growth performance, slaughter characteristics, nutrient digestibility and intestinal microenvironment of broiler chickens. Poult. Sci..

[287] Ahmed S.F., Mofijur M., Rafa N., Chowdhury A.T., Chowdhury S., Nahrin M., Islam A.S., Ong H.C. (2022). Green approaches in synthesising nanomaterials for environmental nanobioremediation: Technological advancements, applications, benefits and challenges. Environ. Res..

[288] Bera S.P., Tank S. (2021). Bioremedial approach of *Pseudomonas stutzeri* SPM-1 for textile azo dye degradation. Arch. Microbiol..

[289] Singh S., Tiwari H., Verma A., Gupta P., Chattopadhaya A., Singh A., Singh S., Kumar B., Mandal A., Kumar R. (2024). Sustainable synthesis of novel green-based nanoparticles for therapeutic interventions and environmental remediation. ACS Synth. Biol..

[290] Harpal A., Pandy P. (2023). Green synthesis of ZnO nanoparticles: A critical review. J. Surv. Fish. Sci..

[291] Mohammadidargah M., Pedram P., Cabrera-Barjas G., Delattre C., Nesic A., Santagata G., Cerruti P., Moeini A. (2024). Biomimetic synthesis of nanoparticles: A comprehensive review on green synthesis of nanoparticles with a focus on *Prosopis farcta* plant extracts and biomedical applications. Adv. Colloid Interface Sci..

[292] Vijayaraghavan K., Ashokkumar T. (2017). Plant-mediated biosynthesis of metallic nanoparticles: A review of literature, factors affecting synthesis, characterization techniques and applications. J. Environ. Chem. Eng..

[293] Alashkar A.A., Mahmoud H.A., Husseien N.F., Mahmoud A.E.-M. (2024). Nanotechnology as a modern technique to impart both natural and synthetic fabrics anti-viral, anti-bacterial and water-repellent properties. J. Text. Color. Polym. Sci..

[294] Dawwam G.E., Al-Shemy M.T., El-Demerdash A.S. (2022). Green synthesis of cellulose nanocrystal/ZnO bio-nanocomposites exerting antibacterial activity and downregulating virulence toxigenic genes of food-poisoning bacteria. Sci. Rep..

[295] Agunbiade F., Allen E., Oyetibo G. (2024). Kinetics of antibacterial activities of cellulose nanocrystals and their silver-zinc oxide nanocomposites: Application as potential disinfectants. Ife J. Sci..

[296] Qu B., Xiao Z., Luo Y., Luo Y. (2023). Carboxymethyl cellulose capped zinc oxide nanoparticles dispersed in ionic liquid and its antimicrobial effects against foodborne pathogens. Carbohydr. Polym. Technol. Appl..

[297] Abdalkarim S.Y.H., Yu H.-Y., Song M.-L., Zhou Y., Yao J., Ni Q.-Q. (2017). In vitro degradation and possible hydrolytic mechanism of PHBV nanocomposites by incorporating cellulose nanocrystal-ZnO nanohybrids. Carbohydr. Polym..

[298] Ikram M., Imran M., Hayat S., Shahzadi A., Haider A., Naz S., Ul-Hamid A., Nabgan W., Fazal I., Ali S. (2022). MoS 2/cellulose-doped ZnO nanorods for catalytic, antibacterial and molecular docking studies. Nanoscale Adv..

[299] Worku L.A., Tadesse M.G., Bachheti R.K., Bachheti A., Husen A. (2024). Synthesis of carboxylated cellulose nanocrystal/ZnO nanohybrids using Oxytenanthera abyssinica cellulose and zinc nitrate hexahydrate for radical scavenging, photocatalytic, and antibacterial activities. Int. J. Biol. Macromol..

[300] Azizy S. (2014). Preparation and Characterization of Poly(vinyl Alcohol)/chitosan Bio-Nanocomposites Reinforced with Cellulose Nanocrystals, Cellulose Nanocrystals/Zinc Oxide and Cellulose Nanocrystals/Zinc Oxide-Silver Nanoparticles. Ph.D. Thesis.

[301] Baldelli A., Etayash H., Oguzlu H., Mandal R., Jiang F., Hancock R.E., Pratap-Singh A. (2022). Antimicrobial properties of spray-dried cellulose nanocrystals and metal oxide-based nanoparticles-in-microspheres. Chem. Eng. J. Adv..

[302] Amin I., Abdelkhalek A., El-Demerdash A.S., Pet I., Ahmadi M., Abd El-Aziz N.K. (2025). Cellulose Nanocrystal/Zinc Oxide Bio-Nanocomposite Activity on Planktonic and Biofilm Producing Pan Drug-Resistant *Clostridium perfringens* Isolated from Chickens and Turkeys. Antibiotics.

[303] Choi E.-S., Choi C.-H., Jeon J.-H., Kim S.-H., Song H.-J., Yi H., Rhie G.-E., Chung Y.-S. (2025). Dual-Toxin-Producing Clostridium botulinum Strain Isolated from a Foodborne Botulism Case in Korea: Genomic and Functional Insights. Toxins.

[304] Priya G.B., Srinivas K., Shilla H., Milton A.A.P. (2023). High prevalence of multidrug-resistant, biofilm-forming virulent *Clostridium perfringens* in broiler chicken retail points in Northeast India. Foods.

[305] Al-Sagan A.A., Abudabos A.M. (2017). Effect of a prebiotic, probiotic and symbiotic on performance of broilers under *Clostridium perfringens* challenge. Thai J. Vet. Med..

[306] Ko H., Goo D., Lee J., Gyawali I., Katha H.R., Lee K.Y., Kim W.K. (2025). Effect of coated organic acids on growth performance, *Clostridium perfringens* colonization, gut integrity and immune response in broilers challenged with subclinical necrotic enteritis. Poult. Sci..

[307] Kerek Á., Szabó Á., Barnácz F., Csirmaz B., Kovács L., Jerzsele Á. (2025). Antimicrobial Susceptibility and Toxin Gene Profiles of Commensal *Clostridium perfringens* Isolates from Turkeys in Hungarian Poultry Farms (2022–2023). Antibiotics.

[308] Rajput D.S., Zeng D., Khalique A., Rajput S.S., Wang H., Zhao Y., Sun N., Ni X. (2020). Pretreatment with probiotics ameliorate gut health and necrotic enteritis in broiler chickens, a substitute to antibiotics. AMB Express.

[309] Xu H., Gong L., Guo Y. (2025). Integrated transcriptomics and metabolomics reveal multi-target mechanisms of tannins against *Clostridium perfringens* and necrotic enteritis. J. Anim. Sci. Biotechnol..

